# MMP1-induced NF-κB activation promotes epithelial–mesenchymal transition and sacituzumab govitecan resistance in hormone receptor-positive breast cancer

**DOI:** 10.1038/s41419-025-07615-y

**Published:** 2025-04-26

**Authors:** Letian Chen, Yinghuan Cen, Keyang Qian, Wang Yang, Wenbin Zhou, Yaping Yang

**Affiliations:** 1 Guangdong Provincial Key Laboratory of Malignant Tumor Epigenetics and Gene Regulation, Sun Yat-sen Memorial Hospital, Sun Yat-sen University, Guangzhou, China; 2 Breast Tumor Center, Sun Yat-sen Memorial Hospital, Sun Yat-sen University, Guangzhou, China; 3 Department of Oncology, The Affiliated Hospital of Jiangnan University, Wuxi, Jiangsu China; 4 Wuxi Medical College, Jiangnan University, Wuxi, China; 5 Division of Breast Surgery, Department of General Surgery, Shenzhen People’s Hospital (The Second Clinical Medical College, Jinan University; The First Affiliated Hospital, Southern University of Science and Technology), Shenzhen, China

**Keywords:** Breast cancer, Prognostic markers

## Abstract

Sacituzumab govitecan (SG), a novel antibody-drug conjugate (ADC), shows promise in the treatment of breast cancer (BC); however, drug resistance limits its clinical application. Matrix metalloproteinase 1 (MMP1), which is overexpressed in many tumor types, plays a key role in tumor metastasis and drug resistance. The involvement of MMP1 in SG resistance in metastatic hormone receptor-positive (HR + ) BC has not been previously reported. In this study, we employed various in vitro and in vivo approaches to investigate the role of MMP1 in SG resistance in BC. MMP1 expression was manipulated in different BC cell lines through lentiviral transfection and small interfering RNA techniques. Key methodologies included Western blot, quantitative reverse transcription PCR, and RNA sequencing to assess marker expression and identify differentially expressed genes. Functional assays were conducted to evaluate cell viability, proliferation, invasion, and migration. In vivo, a cell-derived xenograft model in nude mice was utilized to assess tumor growth and drug response. Bioinformatics analyses further explored MMP1 expression and its clinical relevance across different cancer types. Our findings indicate that MMP1 is overexpressed by approximately 30-fold in HR + BC tissues and is associated with poorer prognosis among HR + BC patients. Furthermore, our analysis reveals that HR + BC with high MMP1 expression displays resistance to SG, supporting the hypothesis that MMP1 plays a key role in regulating ADC resistance. Mechanistic studies demonstrate that MMP1 can activate the NF-κB pathway, which subsequently influences the epithelial–mesenchymal transition, thereby contributing to SG resistance. Ultimately, our research underscores the potential of MMP1 as a therapeutic target and biomarker, facilitating personalized treatment strategies that could enhance patient outcomes in BC therapy.

## Introduction

Breast cancer (BC) is the most prevalent malignancy among women worldwide, with the highest proportion being hormone receptor-positive (HR + ) human epidermal growth factor 2-negative (HER2-), accounting for approximately 70% of all cases [1]. Endocrine therapy remains the primary treatment for HR + /HER2- BC; however, drug resistance is universal in patients with advanced disease. Recently, the combination of cyclin-dependent kinase (CDK) 4/6 inhibitors with endocrine therapy has opened a new horizon for this subtype of patients [2]. CDK4/6 inhibitors halt the cell cycle and inhibit cancer cell proliferation by selectively blocking the CDK4 and CDK6 proteins. They can significantly improve the prognosis of HR + /HER2- BC [2]. However, for patients who present resistance to this strategy, the therapeutic landscape becomes extremely limited, with chemotherapy being the only subsequent option available [3]. Unfortunately, chemotherapy in BC usually prolongs overall survival (OS) by only 3–6 months in metastatic cases and is poorly tolerated, with up to 50% of older patients experiencing severe toxicity, resulting in significant quality of life reductions due to adverse effects [4]. The identification of new targets, such as additional tumor-associated antigens or signaling pathways, could greatly expand the therapeutic spectrum, particularly for patients who are unresponsive to existing targets such as trophoblast cell-surface antigen 2 (Trop2). By focusing on targets related to existing resistance mechanisms, such as those involved in drug transport, it is possible to enhance intracellular drug accumulation, thereby overcoming resistance due to impaired drug transport [5]. Furthermore, the potential for combination therapies is significantly increased with the advent of new targets. Simultaneous targeting of multiple pathways can improve therapeutic outcomes and reduce the likelihood of resistance development [6].

Antibody-drug conjugates (ADCs) represent a novel delivery system for cytotoxic agents in cancer treatment. Sacituzumab govitecan (SG) is a first-in-class Trop2-targeted ADC. It consists of the active metabolite of irinotecan, SN-38—a topoisomerase I (TOP1) inhibitor—covalently linked to the humanized anti-Trop2 monoclonal antibody hRS7 IgG1κ via a hydrolyzable CL2A linker [7]. Trop2, a transmembrane calcium signal transducer, is highly expressed in HR + /HER2- and triple negative breast cancer (TNBC) and is connected with poor prognosis [8]. The phase I/II IMMU-132-01 study has demonstrated preliminary efficacy of SG in previously treated HR + /HER2- metastatic BC patients, showing encouraging overall response rate (ORR), median progression-free survival (mPFS), and median OS [9].

However, despite its clinical benefits, some patients may develop resistance, with a mPFS of approximately 6 months [10]. Several hypotheses regarding resistance to SG have been proposed. Some researchers suggest that Trop2 expression may vary among individuals with HR + /HER2- BC, with some patients exhibiting low or absent Trop2 expression, potentially leading to reduced efficacy of SG [11]. A single-target approach may prove inadequate to effectively address the complex mechanisms of resistance. Resistance involves not only a reduction in target expression but also factors such as impaired drug transport, compromised lysosomal function, and payload-related resistance [12]. This suggests that even when the target remains present, alterations in these additional factors can result in therapeutic failure. Furthermore, specific mutations, such as those in the *TACSTD2* gene, could substantially influence the subcellular distribution of Trop2, thereby altering the antibody’s ability to bind to its antigen. Such mutations can arise during treatment, substantially diminishing the therapeutic efficacy of the original target [13]. The challenge of resistance underscores the complexity of tumor biology and highlights the necessity for a multifaceted therapeutic approach for patients with HR + /HER2- BC.

The extracellular matrix (ECM) is increasingly recognized as a key regulator in BC, showing significant changes in composition and organization throughout cancer progression compared to its homeostatic state in healthy breast tissue [14]. These alterations in ECM can influence tumor behavior and therapeutic response. Proteases such as matrix metalloproteinases (MMPs) are vital for ECM homeostasis and play crucial roles within the tumor microenvironment by facilitating ECM degradation in both normal physiological processes and cancer progression [15, 16]. Elevated expression of matrix metalloproteinase 1 (MMP1) has been associated with poor disease-free survival (DFS) and OS in patients with invasive breast carcinoma [17]. MMP1 has been extensively studied for its role in drug resistance across various cancers. Research indicates that MMP1 contributes to resistance by influencing cell signaling, modifying the tumor microenvironment, and altering ECM composition [18, 19]. By degrading ECM components, MMP1 releases growth factors and signaling molecules that activate pathways such as MAPK/ERK and PI3K/AKT, promoting cell survival and resistance to therapy [20]. In adriamycin-resistant MCF-7 cells, MMP1 expression is significantly higher than in parental cells, partly due to the upregulation of extracellular MMP inducers [21]. Additionally, in tamoxifen-resistant MCF-7 cells and tissues, hypomethylation and overexpression of MMP1 suggest its involvement in resistance to endocrine therapy [22]. However, the precise role of MMP1 in BC development and resistance to specific therapies remains unclear.

Studies indicate that MMP1 may significantly contribute to the facilitation of epithelial–mesenchymal transition (EMT), a critical step in the metastatic cascade of BC. It involves the loss of epithelial cell polarity and intercellular adhesion, thereby facilitating the transition towards a more invasive phenotype [23]. This phenotypic change is characterized by alterations in marker expression, including downregulation of E-cadherin and upregulation of vimentin. Although EMT is partially reactivated in different types of cancer, it often does not reach its full extent in tumor cells, resulting in the lack of end-stage markers such as vimentin. In BC, this transition is accompanied by the induction of ECM proteins, including fibrous collagen, fibronectin, specific laminins, and proteoglycans. These changes enhance the metastatic potential of cancer cells [24]. The precise role of MMP1 in regulating these EMT processes and ECM remodeling warrants further investigation to elucidate its potential as a therapeutic target in BC metastasis.

Expanding on the recognized role of MMP1 in driving the EMT and its impact on BC progression, this study aims to clarify the specific contributions of MMP1 to resistance against SG. To achieve this, we employed a multifaceted approach that integrates in vitro, in vivo, and bioinformatics analyses. Specifically, we conducted RNA sequencing and a comprehensive analysis of The Cancer Genome Atlas (TCGA) datasets to identify differentially expressed genes (DEGs) between BC patients sensitive to SG and those resistant to it. Our findings underscore the significant role of MMP1 in SG resistance. Through both in vitro and in vivo studies, we confirmed the pivotal role of MMP1 in the progression of HR + /HER2- BC and its association with SG resistance. Furthermore, our research suggests that MMP1 may serve as a clinically significant biomarker and therapeutic target for the treatment of HR + /HER2- BC.

## Materials and methods

This study utilized a comprehensive approach to investigate the role of MMP1 in BC progression and resistance to SG. We used different BC cell lines, including both parental and SG-resistant variants, to establish in vitro models. Techniques such as Western blot and quantitative reverse transcription PCR (qRT-PCR) were employed to assess the expression levels of MMP1 and other relevant markers. Additionally, RNA sequencing was conducted to identify differentially expressed genes (DEGs) associated with SG resistance.

To elucidate the function of MMP1, we utilized lentiviral transfection and small interfering RNA (siRNA) techniques to achieve knockdown and overexpression in specific cell lines. Functional assays, including colony formation, cell viability (measured using the CCK-8 assay), proliferation (assessed via EdU incorporation), invasion (evaluated through transwell assays), and migration (measured using wound scratch and transwell assays), were conducted to evaluate the impact of MMP1 on cancer cell characteristics.

In vivo, we employed a cell-derived xenograft (CDX) model in nude mice to assess tumor growth and response to SG treatment. Furthermore, bioinformatics analyses of publicly available databases were performed to explore MMP1 expression across various cancer types and its clinical implications. This multifaceted methodology facilitates a thorough investigation of MMP1’s significance in BC treatment resistance, potentially informing future therapeutic strategies.

### Cell lines and cell culture

In this study, the BC cell lines MCF-7, T47D, and MDA-MB-231 were selected due to their representation of distinct BC subtypes. MCF-7 and T47D are estrogen receptor-positive (ER + ) cell lines commonly utilized to investigate HR + BC. In contrast, MDA-MB-231 represents TNBC, which is characterized by its aggressive nature and poor prognosis, making it a critical model for research on metastasis and drug resistance. All cell lines were employed to explore the potential differences in mechanisms of SG resistance between TNBC and HR + BC.

The parental cell lines (MCF-7, T47D, and MDA-MB-231) were obtained from the American Type Culture Collection (ATCC, Rockville, MD, USA). SG-resistant cell lines (MCF-7-Re, MDA-MB-231-Re, and T47D-Re) were generated using a conventional stepwise method that gradually increased the concentration of SG over a period of 12 months [25].

MCF-7 and MDA-MB-231 cells were cultured in Dulbecco’s Modified Eagle’s Medium (DMEM) (Gibco, USA), while T47D cells were cultured in RPMI-1640 medium (Gibco, USA) supplemented with 10% fetal bovine serum (FBS) (HyClone, USA). The cells were maintained in a humidified atmosphere containing 5% CO_2_ at 37 °C and were passaged every 2–3 days, subsequently sub-cultured at a 1:3 ratio, with three biological replicates.

### Drugs and reagents

SG (cat. no. 1491917-83-9), MMP-1-IN-1 (cat. no. HY-152092), Ilomastat (cat. no. HY-15768) and Pyrrolidinedithiocarbamate ammonium (PDTC) (cat. no. HY-18738) were purchased from MedChemExpress (MCE, New Jersey, USA).

### Western blot

Cells were harvested and lysed using RIPA buffer (89901, Thermo Fisher Scientific) supplemented with 1% protease inhibitors (36978, Thermo Fisher Scientific) at 4 °C for 30 min, with intermittent vortexing. The protein concentration was subsequently determined using a BCA kit (P0011, Beyotime). Proteins were separated by SDS-PAGE under a constant voltage of 120 V for ~90 min, followed by transfer to nitrocellulose membranes at 100 V for 60 min.

The dilution factor for each primary antibody was established based on the manufacturer’s recommendations and preliminary experiments. Specifically, a range of dilutions (1:500, 1:1000, and 1:2000) was measured to identify the optimal concentration with the best signal-to-noise ratio. The incubation time was also optimized through initial trials, with overnight incubation at 4 °C ensuring sufficient binding while minimizing background signal.

Cells were incubated with specific primary antibodies against MMP1 (ab52631, Abcam; rabbit monoclonal antibody specific for human MMP1, 1:1000), vimentin (ab92547, Abcam; rabbit monoclonal antibody recognized for its role in epithelial-mesenchymal transition, 1:1000), N-cadherin (ab76011, Abcam; rabbit polyclonal antibody recognized for its role in EMT, 1:1000), E-cadherin (ab40772, Abcam; rabbit monoclonal antibody validated for use in Western blot, 1:1000), TNF (cat. no. 3707S, Cell Signaling Technology, rabbit monoclonal antibody validated for use in Western blot, 1:1000), pIKKα/β (ab130947, Abcam; rabbit polyclonal antibody specific for phosphorylated IKKα/β, validated for use in Western blot, 1:1000), IKKβ (cat. no. 8943S, CST; rabbit monoclonal antibody validated for use in Western blot, 1:1000), pP65 (cat. no. 3033S, CST; rabbit monoclonal antibody specific for phosphorylated P65, validated for use in Western blot, 1:1000), pIκB (cat. no. 2859S, CST; rabbit polyclonal antibody specific for phosphorylated IκB, validated for use in Western blot, 1:1000), IκB (cat. no. 4812S, CST; rabbit monoclonal antibody validated for use in Western blot, 1:1000), pAKT (cat. no. 4060S, CST; rabbit monoclonal antibody specific for phosphorylated AKT, validated for use in Western blot, 1:1000), and AKT (cat. no. 9272S, CST; rabbit monoclonal antibody validated for use in Western blot, 1:1000) at 4 °C overnight.

Following this, the membranes were incubated with horseradish peroxidase (HRP)-conjugated secondary antibodies at room temperature for 1 h. The bands were visualized using enhanced chemiluminescence. GAPDH (cat. no. 10494-1-AP, Proteintech, rabbit monoclonal antibody validated for use in Western blot, 1:2000) served as an internal control. Relative protein expression was analyzed using ImageJ software by selecting specific regions of interest (ROIs) corresponding to the protein bands. Background correction was carried out by subtracting the mean intensity of a region adjacent to each band. The resulting data were then normalized to the expression levels of housekeeping proteins, ensuring accurate quantification across samples. This process facilitated precise comparisons of protein expression levels across various experimental conditions. The original blot images are included in Supplementary materials.

### Quantitative reverse transcription PCR (qRT-PCR)

Total RNA extraction was performed using the RNA-Quick Purification Kit (ES Science, China) for cultured cells and the RNeasy FFPE Kit (QIAGEN, Germany) for formalin-fixed paraffin-embedded (FFPE) samples. For cell samples, approximately 1 × 10^6^ cells were lysed according to the manufacturer’s protocol, ensuring thorough cell disruption through careful homogenization. In the case of FFPE samples, deparaffinization was achieved using xylene, followed by rehydration in graded alcohols prior to RNA extraction. The quality of RNA was assessed using a NanoDrop spectrophotometer to measure the A260/A280 ratio, while integrity was further confirmed via agarose gel electrophoresis.

Subsequently, complementary DNAs (cDNAs) were synthesized using the Reverse Transcriptase kit (E047-01B, Novoprotein) in accordance with the manufacturer’s instructions. For each reaction, 1 µg of total RNA served as the template in a total reaction volume of 20 µL. The reverse transcription reaction was conducted at 42 °C for 60 min, followed by enzyme inactivation at 85 °C for 5 min. A no-template control (NTC) was included to monitor potential contamination, thereby ensuring the validity of the cDNA synthesis process.

qRT-PCR analysis was carried out using the SYBR Green Pro Taq HS premixed qPCR kit (AG11701-S, Accurate Biotechnology), with a final SYBR Green concentration of 1X in each reaction. Amplification and detection were conducted on the ABI PRISM 7900 (Applied Biosystems) under standard cycling conditions: an initial denaturation step at 95 °C for 30 s, followed by 40 cycles of 95 °C for 5 s and 60 °C for 30 s. Primer efficiency was confirmed by generating a standard curve using serial dilutions of cDNA, which ensured the precise quantification of target gene expression.

The relative gene expression levels were calculated using the 2^-ΔΔCt^ method, with ACTB (β-actin) chosen as the reference gene for normalization. ACTB was selected based on its consistent expression across the experimental conditions, as validated by preliminary experiments. Statistical analysis of the qRT-PCR data was performed using one-way analysis of variance (ANOVA), followed by Tukey’s post-hoc test to evaluate the significance of differences between groups.

The primer sequences, along with their melting temperatures (Tm) and design considerations, are detailed as follows: MMP1 Forward: 5’-AAAATTACACGCCAGATTTGCC-3’ (Tm: 60 °C, designed to span an exon-exon junction to avoid genomic DNA amplification), Reverse: 5’-AAAATTACACGCCAGATTTGCC-3’ (Tm: 60 °C); ACTB Forward: 5’-CATGTACGTTGCTATCCAGGC-3’ (Tm: 58 °C, served as an internal control), Reverse: 5’-CTCCTTAATGTCACGCACGAT-3’ (Tm: 58 °C). Primers of other genes are listed in Table S2.

### RNA sequencing

Total RNA was isolated from both MCF-7-Pa and MCF-7-Re cells using TRIzol reagent, adhering to the manufacturer’s protocol. The quality and quantity of the isolated RNA were evaluated with the Agilent 2100 Bioanalyzer, ensuring high RNA integrity for subsequent applications. For RNA sequencing, RNA libraries were meticulously prepared according to the instructions of the Illumina TruSeq RNA Library Prep Kit. This process involved poly-A selection, fragmentation, cDNA synthesis, and adapter ligation. The prepared libraries were then sequenced on the Illumina NovaSeq 6000 platform, generating paired-end reads of 150 base pairs each, which provided comprehensive coverage of the transcriptome.

To gain insights into the biological processes, gene set enrichment analysis (GSEA) was conducted using GSEA software, version 4.3.1. The statistical significance of enrichment for each pathway was assessed through a hypergeometric test, applying an adjusted *p*-value threshold of 0.05. Furthermore, the Kyoto Encyclopedia of Genes and Genomes (KEGG) pathway enrichment analysis was visualized to enhance the understanding of the underlying biological mechanisms.

### Colony formation assay

For the colony formation assays, 500 cells were seeded per well into 6-well plates and incubated at 37 °C with 5% CO_2_ for 24 h to facilitate cell attachment. The cells were then cultured under these conditions for 10 to 14 days until distinct colonies emerged. Following the incubation period, the colonies were fixed with 4% paraformaldehyde for 10 min at room temperature, and subsequently stained with crystal violet for 30 min to enhance visualization. Excess stain was washed away with distilled water, and colonies containing at least 50 cells were manually counted under a microscope.

### Cell counting kit-8 (CCK-8)

Cells (5 × 10^3^ cells per well) were seeded in 96-well culture plates and incubated for 24 h at 37 °C in a 5% CO_2_ environment to allow for cell adherence. After the initial incubation, predetermined concentrations of the drug were added to the respective cell groups. Subsequently, the cells were treated for 48 h under the same conditions. After treatment, the CCK-8 reagent was prepared by diluting it in serum-free essential medium at a 1:10 ratio. Then, 110 μL of this mixture was added to each well, and the plates were incubated at 37 °C in a 5% CO_2_ environment for an additional 3 to 4 h. Following incubation, the absorbance of each well was quantified using a microplate reader at a wavelength of 450 nm. The absorbance readings were utilized to calculate the half maximal inhibitory concentration (IC50), and dose-response curves were plotted to analyze the cytotoxic effects of the drug on the cells.

### EdU assay

To assess cell proliferation, the EdU assay was performed using the BeyoClick EdU-594 kit (Beyotime, Shanghai, China). Briefly, cells were seeded in 96-well plates at a density of 5 × 10^3^ cells per well and allowed to adhere overnight at 37 °C in a 5% CO_2_ environment. The following day, the culture medium was replaced with fresh medium containing 10 µM EdU, and the cells were incubated for 2 h at 37 °C to facilitate EdU incorporation into newly synthesized DNA. After incubation, the cells were rinsed twice with phosphate-buffered saline (PBS) to remove excess EdU and then fixed in 4% paraformaldehyde for 15 min at room temperature. The cells were rinsed again with PBS and subsequently stained with DAPI for 5 min to visualize cell nuclei. Following a final wash with PBS, fluorescent images were captured using a fluorescence microscope (IX70 Olympus, Tokyo, Japan) to assess cell proliferation.

### Wound scratch

Cells were seeded in triplicate into a 24-well culture plate and allowed to proliferate until they reached 100% confluency. Once confluent, a sterile 100 μL pipette tip was carefully used to create a scratch in the cell monolayer, thereby inducing a wound. Subsequently, the cells were washed twice with PBS to remove any debris or dislodged cells, ensuring a clean wound area. Following the wash, cells were incubated in serum-free medium to inhibit proliferation and focus on cell migration during the wound healing process. Wound closure was monitored over a 24 h period, with images of the wound being periodically captured using an inverted microscope (Olympus, Tokyo, Japan) to track the migration of cells into the wound area.

### Transwell assay

To evaluate cell migration and invasion, cells were seeded into the upper chamber of a transwell insert (Corning Inc, Corning, NY, USA) at a density of 1 × 10^5^ cells per well. For invasion assays, the transwell inserts were pre-coated with Matrigel (BD Biosciences, San Jose, CA, USA) and solidified at 37 °C before cell seeding. For migration assays, the inserts were left uncoated. The transwell system’s lower chamber was filled with medium enriched with 10% FBS, which served as a chemoattractant to enhance cell movement through the membrane. After 24 h of incubation at 37 °C, cells that had migrated or invaded through the membrane were fixed using 4% paraformaldehyde for 15 min at room temperature. Subsequently, the fixed cells were stained with crystal violet for 30 min to enhance visualization. After removing the excess stain by rinsing with PBS, the stained cells on the underside of the membrane were observed and photographed using an inverted microscope (Olympus, Tokyo, Japan).

### Cellular immunofluorescence (IF)

Cells were initially cultured on glass coverslips placed in 24-well plates and allowed to adhere overnight at 37 °C in a 5% CO_2_ environment. Once the cells reached the desired confluency, they were fixed in 4% paraformaldehyde for 20 min at room temperature to preserve cellular structures. Following fixation, the coverslips were rinsed with PBS to remove any residual fixative, and the cells were permeabilized with 0.25% Triton X-100 for 5 min, which facilitated enhanced antibody access to intracellular targets.

Blocking was performed using 5% bovine serum albumin (BSA) for 1 h at room temperature to minimize nonspecific binding. Following the blocking step, the cells were incubated overnight at 4 °C with the primary antibody MMP1 (ab52631, Abcam, 1:100) to ensure optimal binding. The following day, the cells were rinsed thoroughly with PBS to remove any unbound primary antibodies and then incubated with Alexa Fluor 488-conjugated secondary antibodies (ab150077, Abcam, 1:500) for 1 h at room temperature in the dark to prevent photobleaching.

Following the secondary antibody incubation, the cells were counterstained with DAPI for nuclear visualization and mounted onto glass slides using an antifade mounting medium. Finally, the stained cells were observed, and images were captured using a confocal microscope (Leica Microsystems, Milton Keynes, UK), enabling a detailed examination of MMP1 localization within the cells.

### Immunohistochemistry (IHC)

Tumor samples were utilized for IHC staining to evaluate the expression of specific proteins. Initially, paraffin-embedded tissue sections were cut into 4 µm slices and mounted on glass slides. The sections underwent deparaffinization in xylene, followed by rehydration through a graded ethanol series. Antigen retrieval was performed by heating the sections in Tris/EDTA buffer (pH 9.0) using a microwave oven for 15 min, facilitating the exposure of epitopes for antibody binding. After cooling to room temperature, the sections were treated with 3% H_2_O_2_ for 10 min to inhibit endogenous peroxidase activity, followed by a 30-minute incubation with 5% goat serum to block nonspecific binding.

Subsequently, the sections were then incubated overnight at 4 °C with primary antibodies specific to the target molecules, including MMP1 (ab52631, 1:200), E-cadherin (ab40772, 1:200), and Ki67 (ab15580, 1:200). Following the primary antibody incubation, the sections were rinsed with PBS and incubated with HRP-conjugated secondary antibodies for 1 h at room temperature. To visualize the antigen-antibody complexes, the sections were treated with diaminobenzidine (DAB) for color development, followed by counterstaining with hematoxylin to highlight cell nuclei.

The stained slides were thoroughly washed, dehydrated, and mounted for microscopic examination. The sections were observed and photographed using an optical microscope (Olympus, Tokyo, Japan). The staining intensity and the percentage of positive cells were independently evaluated by two pathologists who were blinded to the clinical details, ensuring an unbiased assessment of the IHC results.

### Comet assay

All steps were conducted according to the manufacturer’s protocol (C2041M, Beyotime, China). Briefly, cells were harvested and resuspended in PBS to create a single-cell suspension at a density of 1 × 10^6^ cells/mL. The cell suspension was then mixed with 75 μL of molten agarose provided in the Comet assay kit. Following this, 70 μL of the mixture was carefully pipetted onto a slide pre-coated with a dry layer of agarose, and the mixture was allowed to solidify at 4 °C for 10 min.

Once solidified, the slides were submerged in a lysis solution to disrupt cellular membranes and proteins, leaving the nuclear DNA intact. The slides were then subjected to electrophoresis under alkaline conditions to facilitate the migration of DNA fragments, which were subsequently stained with 20 μL of Propidium iodide solution for 30 min at room temperature to visualize the DNA. Images of the stained DNA were captured using a Zeiss fluorescence microscope. To quantify DNA damage, the DNA tail moment, reflecting the degree of DNA fragmentation, was analyzed using the OpenComet software.

### Tunel assay

The tablets were washed twice with PBS. Fifty μL of TUNEL test solution was added and incubated at 37 °C for 60 min in the dark. During the incubation, the surrounding area was kept moist with water-soaked paper or cotton wool to minimize evaporation of the TUNEL test solution. The samples were then washed three times with PBS.

### Flow cytometric analysis

Flow cytometric analysis of apoptosis was performed using the fluorescein isothiocyanate (FITC) Annexin V Apoptosis Detection Kit I (BD Biosciences, USA), according to the protocol provided.

#### Gene manipulation

##### Lentiviral production and transfection

Lentiviruses were constructed by IGEbio (Guangzhou, China) to knock down MMP1 in resistant cells and to overexpress MMP1 in parental cells. The lentiviral short hairpin RNA (shRNA) sequences used were as follows: shRNA1: 5’-UCAACUUGCCUUUGUCUUCUU-3’, shRNA2: 5’-AAAGAAUUCCUGCAUUUGCUU-3’, and shRNA3: 5’-UCAUCUUCAUCAAAAUGAGCA-3’. A non-targeting shRNA construct served as a negative control, with the following sequence: shRNA-NC: 5’-ACUUAUUUUGUGUUAGAAGAG-3’. The sequence for MMP1 overexpression (denoted as OE) was 5-‘ACTGAGAAAGAAGACAAAGGC-3’, while its negative control (denoted as Vector) was 5’-ATGTCAGTTTGTCAAATACCCCA-3’.

When the cells reached a confluency of 20–35%, they were infected with lentivirus according to the manufacturer’s instructions. At 72 h post-transfection, the cells were selected using puromycin (2 μg/mL, Solarbio, Beijing, China) until resistant colonies emerged. These colonies were expanded, and MMP1 expression was subsequently verified using Western blot. Similarly, the T47D and MDA-MB-231 cell lines were manipulated using the same lentiviral strategy.

##### Short interfering RNA (siRNA) transfection

For siRNA transfection, cells were cultured in 6-well plates until they reached approximately 60–70% confluency. Specific siRNAs were then transfected using Lipo3000 reagent (L3000001, Thermo Fisher Scientific) following the manufacturer’s protocol. The transfection was performed in serum-free medium, and after 6 h, the medium was replaced with complete growth medium. The efficiency of siRNA-mediated interference was verified 48 h post-transfection using qRT-PCR to ensure effective knockdown of the target genes. The siRNA sequences utilized in this study were as follows:

siBCL2A1: 5’- AAUAUAUCCAAAUUCACAGUC-3’,

siWFDC3: 5’- UGAUGAAGGCCUCUAAGUGUA-3’,

siPI3: 5’- UGAUGAAGGCCUCUAAGUGUA-3’,

siCLDN5: 5’- AGAAAAGGAAACUUCAUUCCG-3’,

siMMP1: 5’- UCAACUUGCCUUUGUCUUCUU-3’.

#### Animal experiment

A CDX model was established using BALB/c nude female mice (6 weeks old), obtained from Sun Yat-sen University (Guangzhou, China). All animal experiments were conducted in strict accordance with ethical guidelines and received approval from the Animal Ethics Committee of Sun Yat-sen University. MCF-7-Re cells (1 × 10^6^) from both the MMP1 shRNA and negative control (shNC) groups were subcutaneously injected into the mice.

Seven days post-inoculation, the mice were randomly divided into four groups (5 mice per group) and were intraperitoneally administered SG (24 mg/kg) every 7 days for a duration of 3 weeks. Tumor volume was measured weekly using calipers, with tumor volume calculated using the formula: (length × width^2^)/2. After 28 days, the mice were sacrificed, and the tumors were excised and weighed.

#### Data analysis and bioinformatics

##### Bioinformatical tools used for data analysis

To explore the expression profile of MMP1 across various cancer types, we utilized TIMER (http://timer.comp-genomics.org/timer/), a comprehensive platform dedicated to systematic cancer analysis. TIMER provided data from 1109 BC samples along with their corresponding gene expression profiles [26]. Additionally, we retrieved survival data from two prominent resources: UALCAN (The University of Alabama at Birmingham Cancer Data Analysis Portal; http://ualcan.path.uab.edu/index.html) and KMPLOT (Kaplan-Meier Plotter; https://kmplot.com/analysis/).

To investigate DEGs between tumor and adjacent normal tissues, we employed the gene differential expression module available on TIMER. Statistical significance was determined using the Wilcoxon test. For the RNA sequencing data, MMP1 expression was classified as either low or high based on criteria that included a *p*-value adjustment of less than 0.05 and an absolute log2 fold change (|log2FC | ) greater than 1.

Furthermore, we retrieved OS and DFS data from KMPLOT and UALCAN. To categorize the samples into high- and low-expression groups, we used the median expression level as the threshold. Specifically, samples exceeding the median were classified as the high-expression cohort (Cutoff-High), while those with expression levels below the median were classified as the low-expression cohort (Cutoff-Low). The Cox proportional hazards (PH) model was adopted to calculate the hazard ratio (HR), providing a quantitative assessment of the relationship between MMP1 expression and clinical outcomes.

##### Description of statistical methods

All experimental data were subjected to descriptive statistical analysis, which included the calculation of means, standard deviations, medians, and ranges to summarize the characteristics of the samples and the overall experimental results. For the analysis of DEGs between SG-sensitive and resistant cells, RNA sequencing data were analyzed using the DESeq2 package in R. This statistical method was employed to identify DEGs, with a significance level set at an adjusted *p*-value of <0.05 and a log2FC threshold of ±1.5. Effect sizes (log2FCs) were reported alongside *p*-values to provide a comprehensive understanding of the differential expression.

Subsequent bioinformatics analyses, including GSEA and KEGG pathway enrichment analysis, were performed to explore biological pathways associated with the DEGs. The GSEA software (version 4.3.1) was employed, and statistical significance was evaluated using a hypergeometric test with an adjusted *p*-value threshold of 0.05.

Correlation analysis was conducted to examine the relationship between MMP1 expression and clinical characteristics, such as disease-specific survival (DSS), OS, and DFS. Cox PH models were used to assess the relationship between MMP1 expression levels and survival outcomes, with HRs and 95% confidence intervals (CIs) calculated to quantify the associated risks.

For functional assays, including cell proliferation, migration, and invasion, differences between treatment groups were analyzed using appropriate statistical tests. Depending on the data distribution, either Student’s t-test or one-way ANOVA followed by post-hoc Tukey’s test was utilized to determine statistical significance. A *p*-value of <0.05 was considered statistically significant for all analyses.

## Result

### MMP1 as an ECM-related gene associated with SG resistance in BC cells

**Fig. 1:**
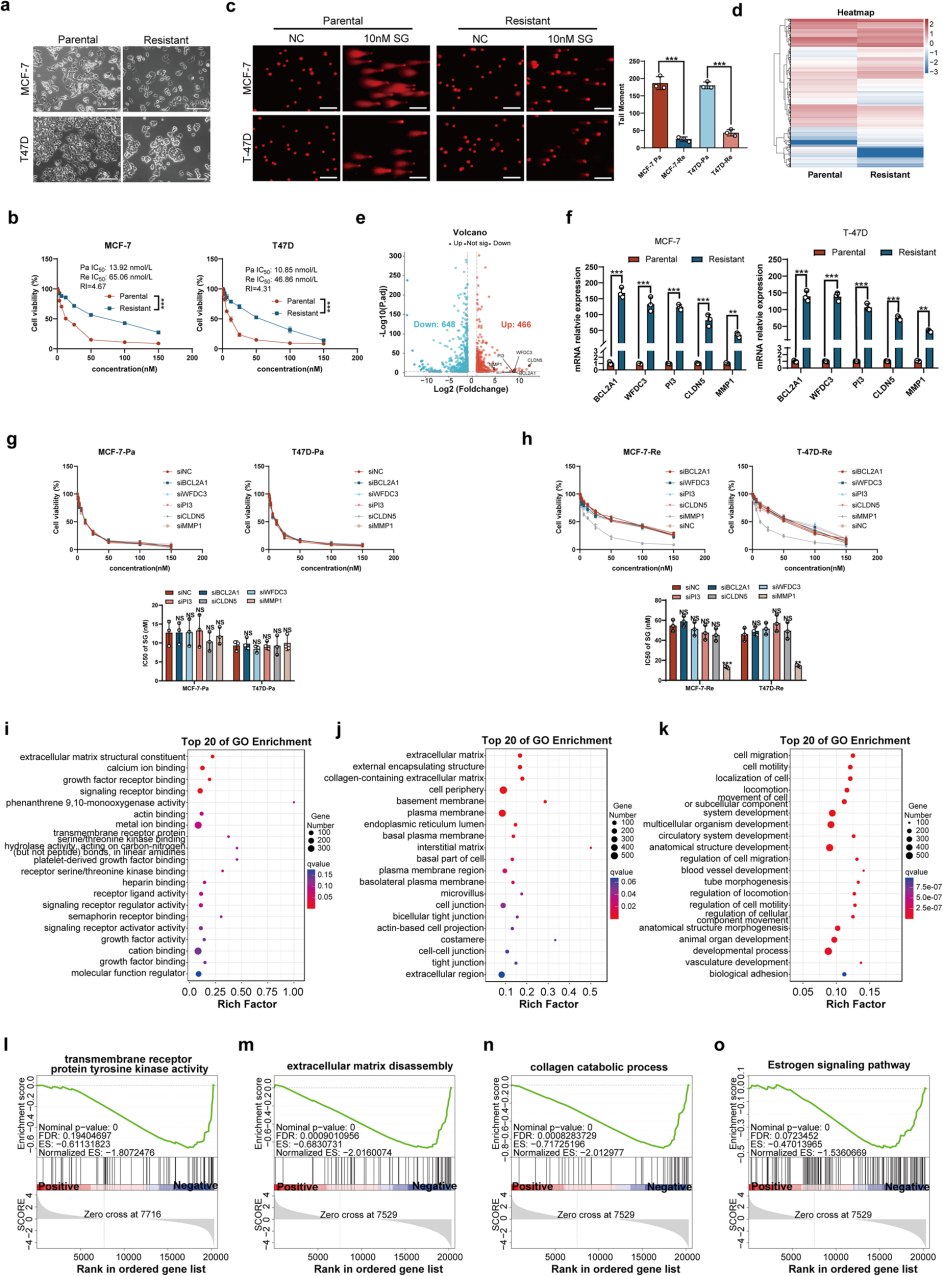
MMP1 as an ECM-related gene associated with SG resistance in BC cells. **a** Cell morphology was observed under a microscope (Scale bar: 100 µm). **b** Cell viability was assessed after 24 h of treatment with 10 nM SG at various concentrations using the CCK-8 assay. **c** Representative fluorescence images and quantification of tail moment in parental and resistant cells. **d** The heatmap displays gene expression in the resistant group compared to the parental group in MCF-7 cells. The colors of the heatmap reflect log2 expression levels of genes. **e** Volcano plots illustrate differential gene expressions in MCF-7-Re compared to MCF-7-Pa cells. Red dots indicate genes with significantly higher expression levels, while green dots represent lower expression levels. **f** qRT-PCR verified expressions of BCL2A1, WFDC3, PI3, CLDN5, and MMP1 between parental and resistant cells. **g** After knockdown of BCL2A1, WFDC3, PI3, CLDN5, and MMP1 separately, the viability of MCF-7-Pa and T47D-Pa was tested at different concentrations of SG using CCK-8 assays. **h** After knockdown of BCL2A1, WFDC3, PI3, CLDN5, and MMP1 separately, the viability of MCF-7-Re and T47D-Re was tested at different concentrations of SG using CCK-8 assays. IC50 values for all groups were calculated using GraphPad and compared using Student’s t-test. **i**–**k** GO enrichment analysis was performed on genes involved in biological processes, cellular components, and molecular functions. **l**–**o** Enrichment of cancer biomarkers based on DEGs between resistant cells and parental cells using GSEA.

To identify key factors related to SG resistance in HR + BC, we first generated SG-resistant cell lines (MCF-7-Re and T47D-Re) through a step-up therapy over a period of 12 months. After induction, we observed distinct differences in cytomorphology between the resistant and parental cells. Unlike the epithelial-like shape observed in the parental group, the resistant cells exhibited a typical mesenchymal-like morphology (Fig. 1a).

According to the IC50 values obtained from the CCK-8 assay, we found that the drug resistance of MCF-7-Re and T47D-Re cells to SG was significantly higher than that of MCF-7-Pa cells (65.06 nmol/L vs. 13.92 nmol/L) and T47D-Pa cells (46.86 nmol/L vs. 10.85 nmol/L), respectively. Specifically, the SG drug resistance index for MCF-7-Re and T47D-Re cells was 4.67-fold and 4.31-fold greater than that of the parental cells (Fig. 1b). The comet assay demonstrated a selective induction of DNA damage upon treatment with SG in the MCF-7-Pa and T47D-Pa cell lines, as evidenced by a significant increase in the tail moment compared to the MCF-7-Re and T47D-Re cell lines (Fig. 1c).

mRNA was extracted from both the parental cell line (MCF-7-Pa) and the resistant cell line (MCF-7-Re) and analyzed using mRNA sequencing to identify DEGs between the two cell lines (Fig. 1d, e). Details of all the DEGs were listed in Table S1. Based on the results, the top five DEGs (BCL2A1, WFDC3, PI3, CLDN5, and MMP1) were selected, and the detailed information was listed in Table 1. The expression of the top five genes were verified in SG-sensitive and SG-resistant cell lines by qRT-PCR (Fig. 1f). We subsequently knocked down the expression of the top five genes and the sensitivity to SG was retested using the CCK-8 assay, and the results demonstrated that no significant change was noticed after knockdown in parental cell lines (Fig. 1g), while in resistant cell lines only the knockdown of MMP1 significantly improved the sensitivity to SG (Fig. 1h).

**Table 1:** Details of the top 5 differential expression genes.

Gene	LogFC	*p*-Value	*q*-Value	Description
BCL2A1	12.17461365	4.77E-19	3.97E-18	BCL2 related protein A1
WFDC3	10.56795608	7.69E-06	2.37E-05	WAP four-disulfide core domain 3
PI3	10.55937709	0.000123	0.000328	peptidase inhibitor 3
CLDN5	9.154818109	2.41E-07	8.63E-07	claudin 5
MMP1	4.65324724	1.04E-11	5.40E-11	matrix metallopeptidase 1

Gene Ontology (GO) enrichment analyses were conducted, revealing significant enrichment in terms such as ECM structural constituent, growth factor receptor binding, signaling receptor binding, cell periphery, cell junction, and cell migration (Fig. 1i, j, and k). Additionally, GSEA revealed enrichment in gene sets associated with transmembrane receptor protein tyrosine kinase activity, ECM disassembly, collagen catabolic processes, and the estrogen signaling pathway (Fig. 1l-o).

In summary, we successfully established SG-resistant cell lines (MCF-7-Re and T47D-Re) that displayed typical mesenchymal-like characteristics morphologically. CCK-8 assays demonstrated that MCF-7-Re and T47D-Re cells showed significantly higher resistance to SG compared to their parental counterparts. Comet assay results indicated that the resistant cells exhibited lower levels of DNA damage than the parental cells. RNA sequencing analysis identified DEGs, with high expression of MMP1 closely associated with SG resistance. These findings illustrate that the knockdown of MMP1 significantly increased the sensitivity of resistant cells to SG, laying the groundwork for future studies on the role of MMP1 in SG resistance.

### High expression of MMP1 in HR + BC tissues and its association with poor prognosis

**Fig. 2:**
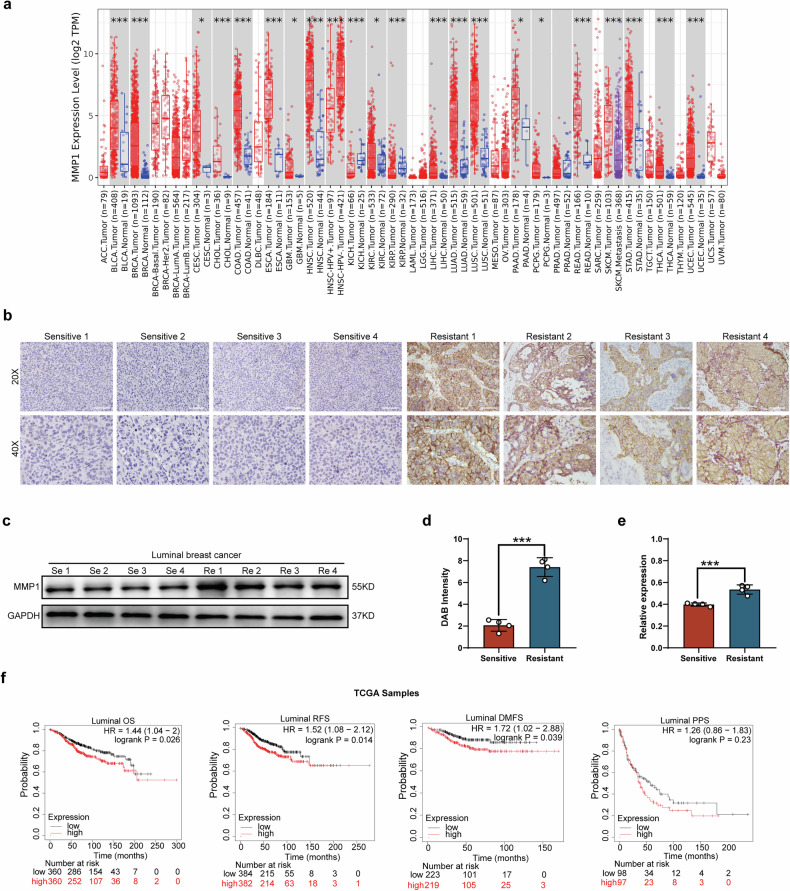
High expression of MMP1 in HR + BC tissues and its association with poor prognosis. **a** MMP1 expression is significantly higher in malignant tissues compared to normal tissues across various cancer types. The TIMER 2.0 database shows that MMP1 is significantly overexpressed in 20 types of malignancies relative to normal tissues. Statistical significance is indicated by asterisks. **b** MMP1 expression levels in SG-resistant and SG-sensitive groups were assessed by IHC using clinical samples from BC patients. **c** Protein levels of MMP1 in clinical samples from SG-resistant and SG-sensitive patients were analyzed by Western blot. **d** Quantitative analysis of panel b is presented in histogram form. **e** Quantitative analysis of panel c is presented in histogram form. **f** Survival analysis of patients with high or low expression of MMP1 in the TCGA database (* *p* < 0.05, ** *p* < 0.01, *** *p* < 0.001).

To characterize the expression level of MMP1 in tumor tissues compared to adjacent nontumor tissues, we utilized the public TIMER database to analyze MMP1 expression across various cancer types. As shown in Fig. 2a, MMP1 was significantly overexpressed in bladder urothelial carcinoma (BLCA), breast cancer (BRCA), cervical squamous cell carcinoma (CESC), cholangiocarcinoma (CHOL), colon adenocarcinoma (COAD), esophageal carcinoma (ESCA), glioblastoma (GBM), head and neck squamous cell carcinoma (HNSC), kidney renal clear cell carcinoma (KIRC), liver hepatocellular carcinoma (LIHC), lung adenocarcinoma (LUAD), lung squamous cell carcinoma (LUSC), prostate adenocarcinoma (PRAD), stomach adenocarcinoma (STAD), thyroid carcinoma (THCA), and uterine corpus endometrioid carcinoma (UCEC) when compared to their corresponding nontumor tissues.

Furthermore, the expression of MMP1 in SG-resistant patients was found to be 3.5 times higher than that in SG-sensitive patients, as demonstrated by IHC staining (Fig. 2b, d). Similarly, Western blot analysis revealed that MMP1 expression in SG-resistant tumors was 1.5 times greater than that in sensitive tumors (Fig. 2c, e). The clinical characteristics of patients based on MMP1 expression levels are tabulated in Table 2.

**Table 2:** Characteristics of different expression level of MMP1.

Characteristics	Low expression of MMP1	High expression of MMP1	*P*-value
*n*	543	544	
Pathologic T stage, *n* (%)			<0.001
T1&T2	433 (39.9%)	476 (43.9%)	
T3&T4	108 (10%)	67 (6.2%)	
Pathologic N stage, *n* (%)			0.003
N0	267 (25%)	249 (23.3%)	
N1&N2	215 (20.1%)	260 (24.3%)	
N3	50 (4.7%)	27 (2.5%)	
Pathologic M stage, *n* (%)			0.118
M0	431 (46.6%)	474 (51.2%)	
M1	6 (0.6%)	14 (1.5%)	
Pathologic stage, *n* (%)			0.053
Stage I	97 (9.1%)	85 (8%)	
Stage II	295 (27.8%)	324 (30.5%)	
Stage III	133 (12.5%)	111 (10.4%)	
Stage IV	5 (0.5%)	13 (1.2%)	
Histological type, *n* (%)			<0.001
Infiltrating Lobular Carcinoma	157 (16%)	48 (4.9%)	
Infiltrating Ductal Carcinoma	322 (32.8%)	454 (46.3%)	
PAM50, *n* (%)			<0.001
LumA	370 (35.3%)	194 (18.5%)	
LumB	82 (7.8%)	124 (11.8%)	
Her2	16 (1.5%)	66 (6.3%)	
Basal	54 (5.2%)	141 (13.5%)	
Age, *n* (%)			0.036
<= 60	284 (26.1%)	319 (29.3%)	
> 60	259 (23.8%)	225 (20.7%)	
Menopause status, *n* (%)			0.845
Pre	113 (11.6%)	117 (12%)	
Post & Peri	372 (38.1%)	374 (38.3%)	

To further confirm the association between MMP1 expression levels and patient survival, we analyzed data from the TCGA database. The results indicated that patients with high MMP1 expression had a significantly shorter OS of 86 months compared to 151 months for patients with low MMP1 expression (*p* < 0.05). Similarly, the recurrence-free survival (RFS) was 76 months for patients with high MMP1 expression versus 113 months for those with low expression (*p* < 0.05). The distant metastasis-free survival (DMFS) was also significantly shorter, at 54 months for high MMP1 expression compared to 123 months for low MMP1 expression (*p* < 0.05). However, the post-progression survival (PPS) did not illustrate a significant difference between the two groups, suggesting that MMP1 could serve as an indicator of poor prognosis in HR + BC patients (Fig. 2f).

Univariate and multivariate Cox analysis, which involved characteristics such as pathological stage, ER status, PR status, HER2 status, histological type, menopause status, and age, was employed to explore crucial prognostic factors. Specifically, Table 3 demonstrates that patients with high MMP1 expression had a higher risk of poor outcomes in terms of DSS, with an HR of 2.034 (95% CI: 1.183–3.498, *p* = 0.010), serving as an independently prognostic factor for HR + BC.

**Table 3:** COX univariate and multivariate analysis of MMP1 and other characteristics.

Characteristics	Total(N)	Univariate analysis		Multivariate analysis	
		Hazard ratio (95% CI)	*P*-value	Hazard ratio (95% CI)	*P*-value
MMP1	1066				
Low	530	Reference		Reference	
High	536	1.771 (1.142–2.748)	**0.011**	2.034 (1.183–3.498)	**0.010**
Pathologic stage	1044				
Stage I & Stage II	790	Reference		Reference	
Stage III & Stage IV	254	3.486 (2.253–5.394)	**<0.001**	3.502 (2.157–5.686)	**< 0.001**
ER status	1019				
Indeterminate & Positive	787	Reference		Reference	
Negative	232	1.764 (1.108–2.808)	**0.017**	1.276 (0.605–2.694)	0.522
PR status	1018				
Indeterminate & Positive	684	Reference		Reference	
Negative	334	1.906 (1.228–2.957)	**0.004**	1.352 (0.665–2.748)	0.405
HER2 status	718				
Indeterminate & Positive	166	Reference			
Negative	552	0.714 (0.358–1.426)	0.340		
Histological type	962				
Infiltrating Ductal Carcinoma	762	Reference		Reference	
Infiltrating Lobular Carcinoma	200	0.472 (0.226–0.986)	**0.046**	0.412 (0.172–0.989)	**0.047**
Menopause status	965				
Pre & Peri	268	Reference			
Post	697	1.552 (0.867–2.780)	0.139		
Age	1066				
<= 60	592	Reference		Reference	
> 60	474	1.446 (0.942–2.222)	0.092	2.005 (1.212–3.319)	**0.007**

The bold values indicate statistically significant differences (*P* < 0.05) compared to the other group

In summary, these results indicate that MMP1 was highly expressed in HR + BC and was associated with poorer prognosis.

### Role of high MMP1 expression in enhancing migration, invasion, and proliferation of HR + BC cells

**Fig. 3:**
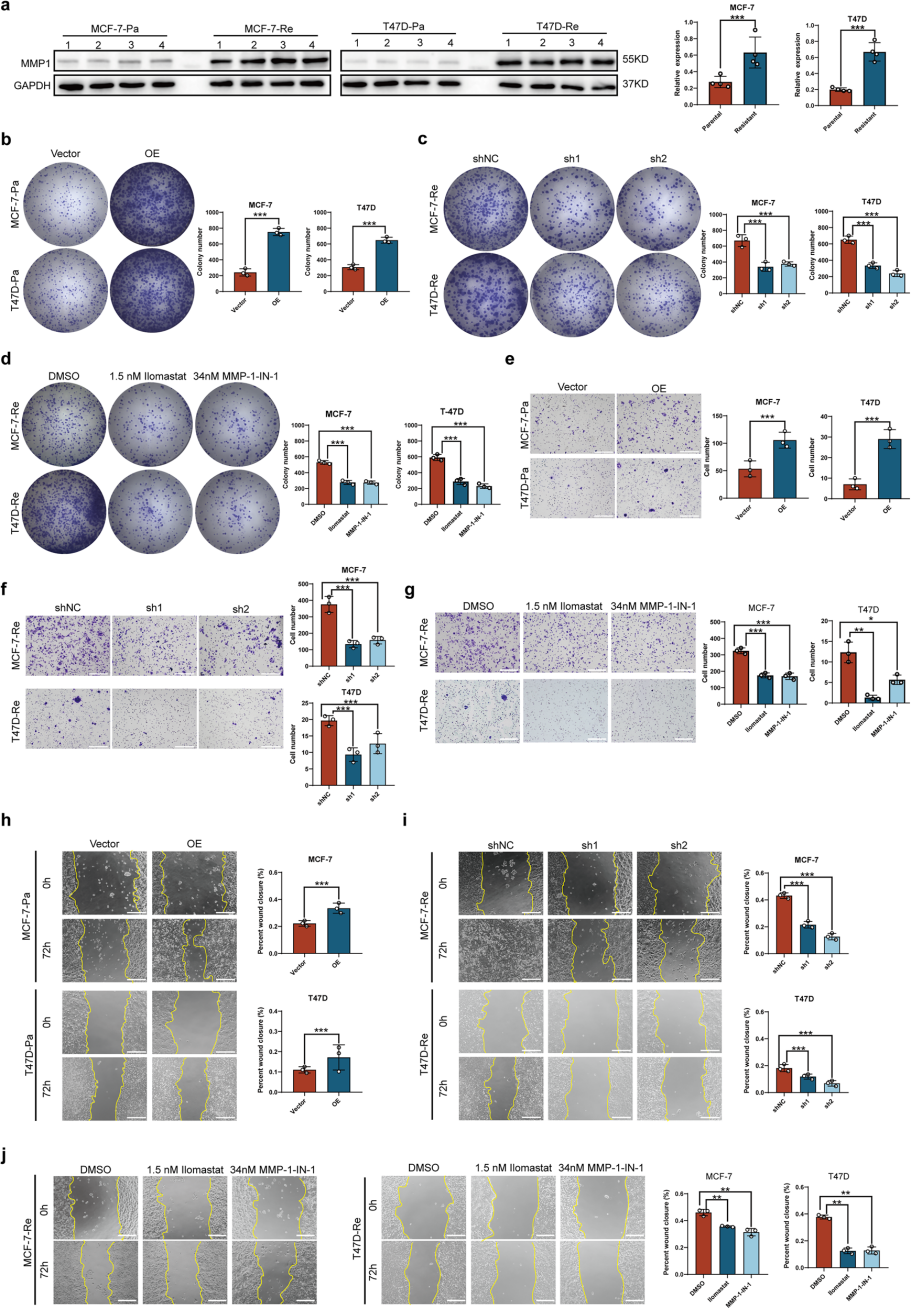
Role of high MMP1 expression in enhancing migration, invasion, and proliferation of HR + BC cells. **a** MMP1 protein expression levels in parental and resistant cells were measured by Western blot analysis. GAPDH served as an internal control. **b** Cell proliferation assessed via colony formation assay following MMP1 overexpression in parental cells. **c**, **d** Cell proliferation assessed via colony formation assay following MMP1 knockdown and inhibition in resistant cells. **e** Cell invasion assessed via transwell assays following MMP1 overexpression in parental cells. **f**, **g** Cell invasion assessed via transwell assays following MMP1 knockdown and inhibition in resistant cells. **h** Cell migration assessed via wound scratch assay following MMP1 overexpression in parental cells. **I**, **j** Cell migration assessed via wound scratch assay following MMP1 knockdown and inhibition in resistant cells (Scale bar: 100 µm). Data are from three independent experiments. *p*-values were determined using a two-tailed unpaired Student’s t-test. * *p* < 0.05, ** *p* < 0.01, *** *p* < 0.001.

To investigate the role of MMP1 in SG resistance among HR + BC cells in vitro, we utilized two different HR+ cell lines, MCF-7-Re and T47D-Re, and performed Western blot analyses to assess their MMP1 expression levels. The results indicated that MCF-7-Re and T47D-Re cells exhibited higher MMP1 expression compared to their parental counterparts, MCF-7-Pa and T47D-Pa, respectively (Fig. 3a).

The two SG-resistant cell lines demonstrated enhanced proliferative and migratory capabilities compared to the parental cell lines, as evidenced by colony formation assays, EdU assays, transwell migration and invasion experiments, and wound scratch assays (Fig. S1a–d). Subsequently, we employed transfected plasmids carrying the *MMP1* gene to increase MMP1 expression in SG-sensitive cells and shRNA to reduce MMP1 expression in SG-resistant cells. Western blot analysis confirmed that MMP1 was significantly overexpressed in MCF-7-Pa and T47D-Pa cells, while its expression was decreased in MCF-7-Re and T47D-Re cells (Fig. S1e, f).

Colony formation assays revealed that MMP1 overexpression significantly increased the number of colonies in MCF-7-Pa and T47D-Pa cells, whereas silencing MMP1 or using MMP1-inhibitors resulted in a marked decrease in the number of colonies in MCF-7-Re and T47D-Re cells (Fig. 3b–d). These findings were consistent with the results from cell invasion experiments (Fig. 3e–g) and wound scratch assays (Fig. 3h–j).

In the MDA-MB-231 cell line, we also induced a resistant variant (Fig. S2a) and investigated MMP1 expression in both the parental and resistant cell lines using reverse transcription quantitative PCR (qRT-PCR) and Western blot analysis. However, the results indicated no significant difference in MMP1 expression between the two cell lines (Fig. S2b–d).

Overall, these data suggest that MMP1 played a crucial role in enhancing cell migration and invasion and may contribute to the resistance of HR + BC cells to SG (all *p* < 0.05).

### MMP1-induced EMT and proliferation via NF-κB pathway activation in BC cells

Our previous findings indicated that SG resistance in HR + BC cells was significantly correlated with EMT and ECM organization, likely mediated by MMP1. Researchers have increasingly recognized the involvement of EMT in chemoresistance, acknowledging its potential role as a contributing factor to the resistance exhibited by tumors against chemotherapy and radiotherapy [27]. Therefore, we further investigated the hypothesis that MMP1 may promote resistance to SG through mechanisms dependent on EMT by employing RNA sequencing analysis to elucidate the signaling pathways orchestrated by MMP1.

**Fig. 4:**
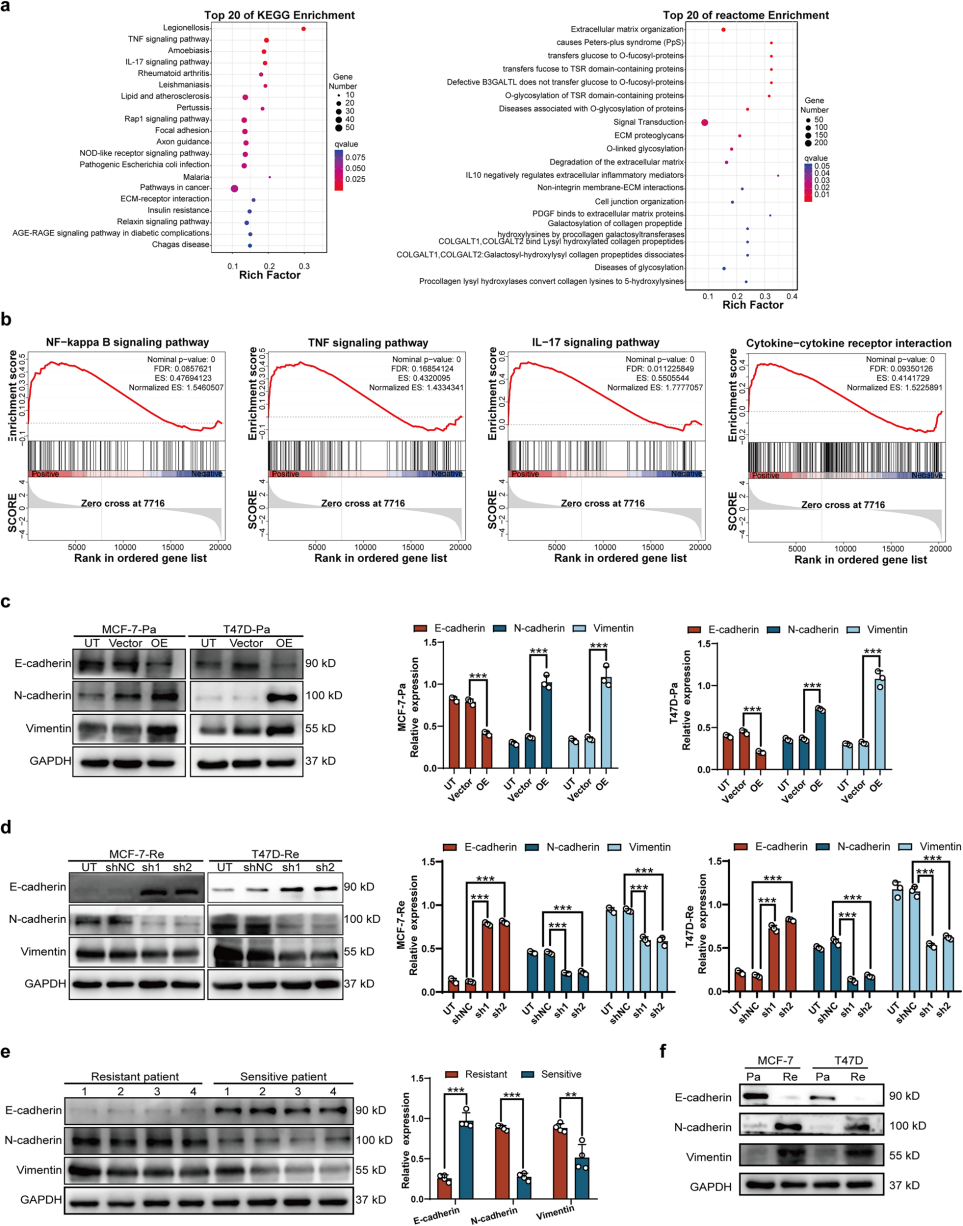
MMP1-induced EMT and proliferation via NF-κB pathway activation in BC cells. **a** KEGG pathway and reactome enrichment analyses of the DEGs, highlighting the top 20 related pathways expressed in MCF-7-Re and T47D-Re cells. **b** Transcriptome data analyzed using GSEA indicated that MMP1 expression levels were positively correlated with metastasis and the NF-κB, TNF, IL-17, and cytokine-cytokine receptor interaction signaling pathways. **c** MCF-7-Pa and T47D-Pa cells were transfected with an empty vector or MMP1 for 48 h, and the expression of related EMT proteins (N-cadherin, E-cadherin, and vimentin) was measured by Western blot. **d** SG-resistant BC cells were transfected with MMP1 shRNA and the corresponding control shRNA for 48 h, and the expression levels of different EMT proteins (N-cadherin, E-cadherin, and vimentin) were measured by Western blot. **e, f** The samples of patients and cell lines of resistant to SG and sensitive to SG were obtained, and the expression levels of different EMT proteins (N-cadherin, E-cadherin, and vimentin) were measured by Western blot. GAPDH served as an internal control. Corresponding quantitative data for N-cadherin, E-cadherin, and vimentin are shown. Data are from three independent experiments. UT untreated. * *p* < 0.05, ** *p* < 0.01, *** *p* < 0.001.

KEGG enrichment analysis revealed the top 20 related pathways, including the TNF signaling pathway, enriched in MCF-7-Re cells (Fig. 4a, left). Additionally, reactome enrichment analysis indicated a significant elevation in ECM organization (*p* < 0.05, FC = 2.5), suggesting that both the TNF signaling pathway and ECM organization may contribute to SG resistance (Fig. 4a, right). Furthermore, GSEA demonstrated that MMP1 expression levels were positively correlated with the NF-κB signaling pathway (normalized ES 0.48, FDR 0.09), TNF (normalized ES 1.43, FDR 0.17), IL-17 signaling pathway (normalized ES 1.78, FDR 0.01), and cytokine-cytokine receptor interactions (normalized ES 1.52, FDR 0.09) (Fig. 4b). Both TNF and IL-17 are known to promote metastasis by regulating the NF-κB signaling pathways within the cytokine-cytokine receptor interaction process, as reported in previous studies [28].

To evaluate the correlation between EMT and MMP1 expression in HR + BC cells, we measured the expression of EMT markers (N-cadherin, E-cadherin, and vimentin) using Western blot analysis after either knocking down or overexpressing MMP1. The results revealed that MMP1 overexpression led to increased levels of N-cadherin and vimentin, along with a decrease in E-cadherin expression (Fig. 4c). Conversely, MMP1 knockdown in SG-resistant cell lines resulted in elevated E-cadherin levels and reduced expression of N-cadherin and vimentin (Fig. 4d). Consistent with these findings, the expression of N-cadherin and vimentin was relatively higher, while E-cadherin was relatively lower in BC tissues from SG-resistant patients compared to those from SG-sensitive patients (Fig. 4e). In cell line samples, expression of N-cadherin and vimentin was relatively higher, while E-cadherin was relatively lower in resistant group compared to parental one (Fig. 4f)

**Fig. 5:**
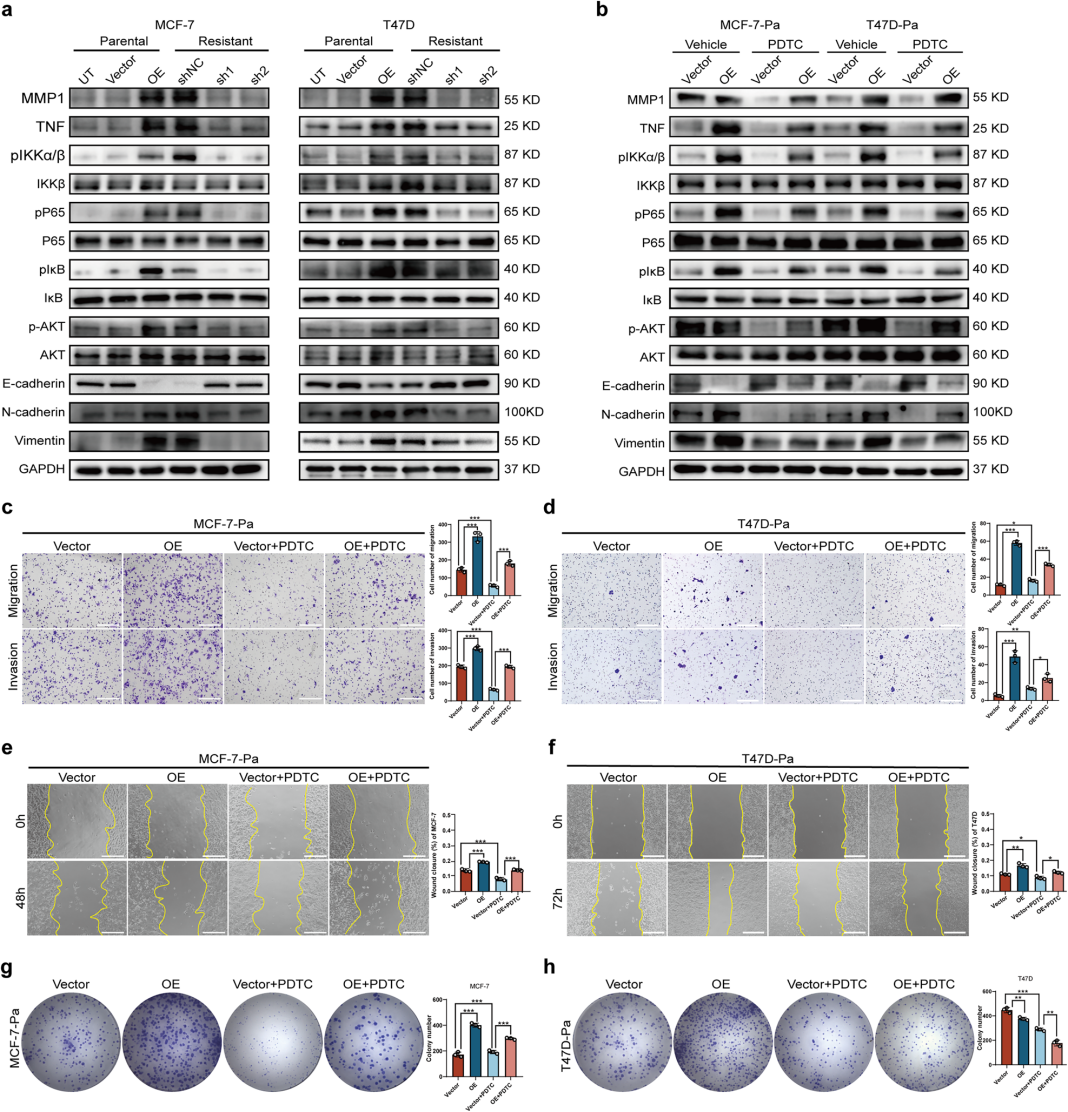
MMP1 promotes EMT and proliferation by activating NF-κB pathway in BC cells. **a** Effects of MMP1 overexpression and knockdown in parental and resistant cell lines on the NF-κB pathway and EMT markers. MMP1, TNF, pIKKα/β, IKKβ, pP65, P65, pIκB, IκB, AKT, p-AKT, and EMT-related protein markers were detected by Western blot. **b** MCF-7-Pa and T47D-Pa cells were transfected with a vector or MMP1, followed by treatment with the NF-κB inhibitor PDTC for 24 h. The expression levels of TNF, MMP1, pIKKα/β, IKKβ, pP65, P65, pIκB, IκB, AKT, p-AKT, and EMT-related protein markers were assessed via Western blot. GAPDH served as an internal control. **c**, **d** Transwell assays were conducted respectively in MCF-7-Pa and T-47D-Pa cells transduced with a vector or MMP1 to evaluate the effects of MMP1 or the NF-κB inhibitor PDTC on cell migration and invasion. **e**, **f** Wound scratch assays were performed respectively in MCF-7-Pa and T-47D-Pa cells transduced with a vector or MMP1 to assess the effects of MMP1 or the NF-κB inhibitor PDTC on cell migration. **g**, **h** Colony formation assays were utilized respectively to detect differences in proliferation ability under MMP1 overexpression with or without PDTC treatment in MCF-7-Pa and T-47D-Pa. UT, untreated. * *p* < 0.05, ** *p* < 0.01, *** *p* < 0.001.

Then, we explored the molecular changes associated with MMP1-mediated EMT in BC cells. The GSEA results indicated a positive correlation between MMP1 expression and the NF-κB pathway (normalized ES 0.48, FDR 0.09), prompting us to examine proteins involved in the NF-κB pathway. We found that MMP1 knockdown decreased the phosphorylation levels of IKKα/β, P65, IκB, and AKT, while elevated phosphorylation levels of these proteins were observed in MMP1-overexpressing cells. Meanwhile, expression of N-cadherin and vimentin was reduced while E-cadherin was elevated in knockdown group (Fig. 5a). To confirm whether TNF/NF-κB activation plays a crucial role in MMP1-induced EMT, we applied the pathway inhibitor PDTC. Our data demonstrated that PDTC treatment significantly reduced the levels of pIKKα/β, pP65, pIκB, and p-AKT in MMP1-overexpressing BC cells. Additionally, PDTC treatment significantly reversed the protein expression of N-cadherin, E-cadherin, and vimentin induced by MMP1 (Fig. 5b).

Moreover, inhibition of the NF-κB pathway by PDTC in MMP1-overexpressing BC cells weakened cell migration, invasion, and proliferation, as evidenced by transwell assays (Fig. 5c, d) and wound scratch assays (Fig. 5e, f). Compared to the control group of MCF-7 and T47D parental cells, MMP1 overexpression enhanced the colony formation ability of BC cells. Notably, PDTC treatment decreased the colony number of MMP1-overexpressing BC cells (Fig. 5g, h). As a control, we compared changes in IC50 values, DNA damage levels, proliferation, invasion and migration abilities before and after PDTC treatment in both MDA-MB-231-Pa and -Re cell lines to illustrate the unique role of the NF-κB pathway in SG resistance in HR + BC (Fig. S2e–j).

In summary, these data indicate that MMP1 promoted EMT and proliferation by activating the NF-κB pathway in HR + BC.

### MMP1 inhibition and its role in overcoming SG resistance in vitro and in vivo

**Fig. 6:**
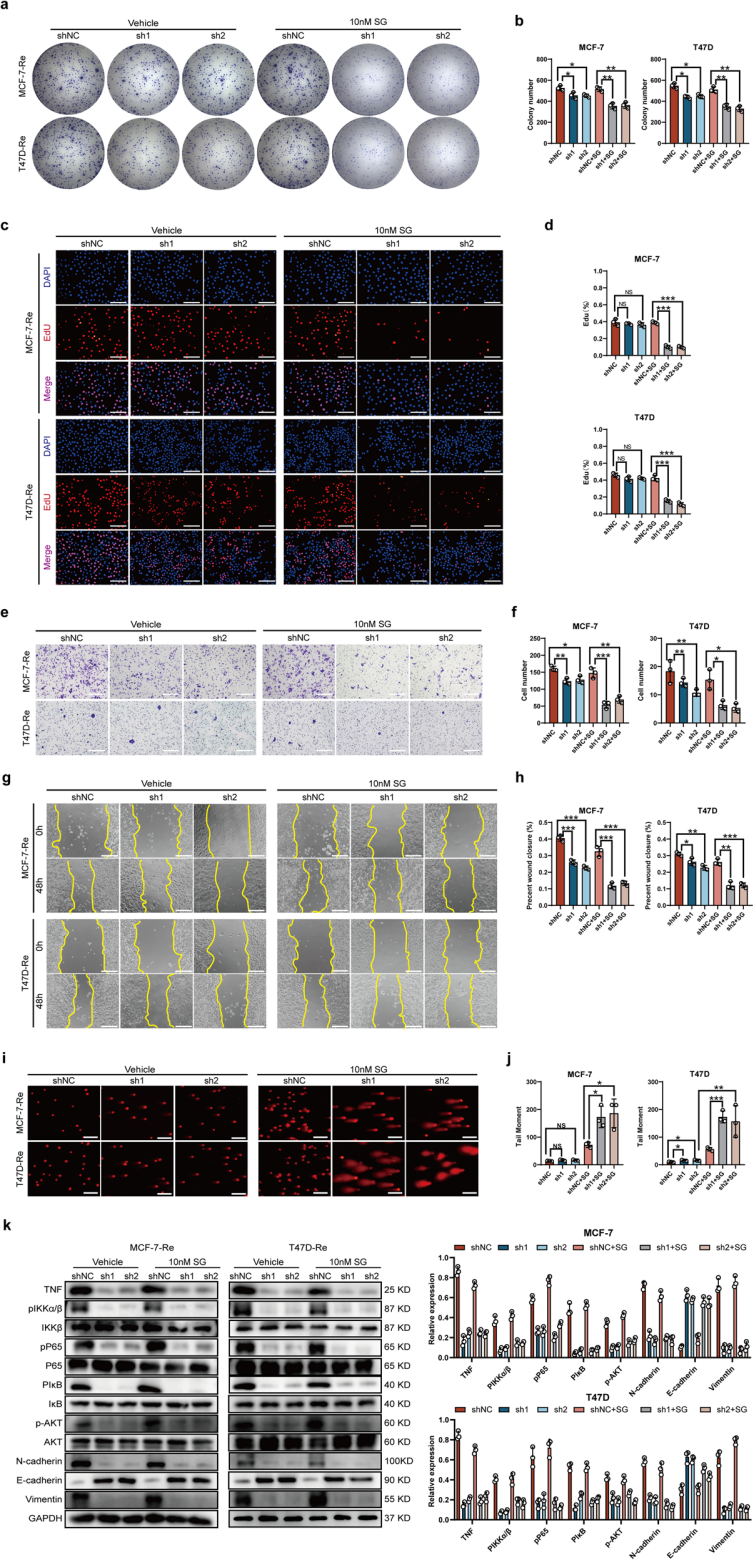
MMP1 inhibition enhances SG sensitivity in SG-resistant BC cells in vitro. **a**, **b** Representative images and corresponding quantitative analysis of colony formation assays from MCF-7-Re and T47D-Re cells treated with MMP1 shRNA or its control for 48 h, followed by treatment with 10 nM SG for 24 h. **c**, **d** Representative images and corresponding quantitative analysis of EdU assays from MCF-7-Re and T47D-Re cells treated with MMP1 shRNA or its control for 48 h, followed by treatment with 10 nM SG for 24 h. **e, f** Representative images and corresponding quantitative analysis of transwell assays from MCF-7-Re and T47D-Re cells treated with MMP1 shRNA or its control for 48 h, followed by treatment with 10 nM SG for 24 h. **g**, **h** Representative images and corresponding quantitative analysis of wound scratch assays from MCF-7-Re and T47D-Re cells treated with MMP1 shRNA or its control for 48 h, followed by treatment with 10 nM SG for 24 h. **i**, **j** Representative fluorescence images and quantification of tail moments in MCF-7-Re and T47D-Re cells treated with MMP1 shRNA or its control for 48 h, followed by treatment with 10 nM SG for 24 h. **k** Representative images and corresponding quantitative analysis of expression of NF-κB pathway and EMT markers from MCF-7-Re and T47D-Re cells treated with MMP1 shRNA or its control for 48 h, followed by treatment with 10 nM SG for 24 h. * *p* < 0.05, ** *p* < 0.01, *** *p* < 0.001.

To examine the effect of MMP1 on SG sensitivity in HR + BC, we assessed the therapeutic impact of SG before and after MMP1 inhibition, both in vitro and in vivo. In vitro, our results indicated that following MMP1 inhibition in MCF-7-Re or T47D-Re cells, treatment with 10 mM SG led to a reduction in colony formation ability by approximately 50% (Fig. 6a, b). Additionally, we observed decreased proliferation (Fig. 6c, d), invasion (Fig. 6e, f), and migration abilities (Fig. 6g, h), along with increased DNA damage as indicated by the tail moment (Fig. 6i, j). Notably, silencing MMP1 inhibited NF-κB pathway and EMT-related markers with or without SG treatment, suggesting that the alteration of NF-κB pathway and EMT-related markers was triggered by MMP1 rather than SG treatment (Fig. 6k). These findings suggest that silencing MMP1 not only enhanced the cytotoxic effects of SG on MCF-7-Re and T47D-Re cells but also inhibited NF-κB pathway and EMT, indicating that MMP1 may serve as a potential target for overcoming SG resistance.

**Fig. 7:**
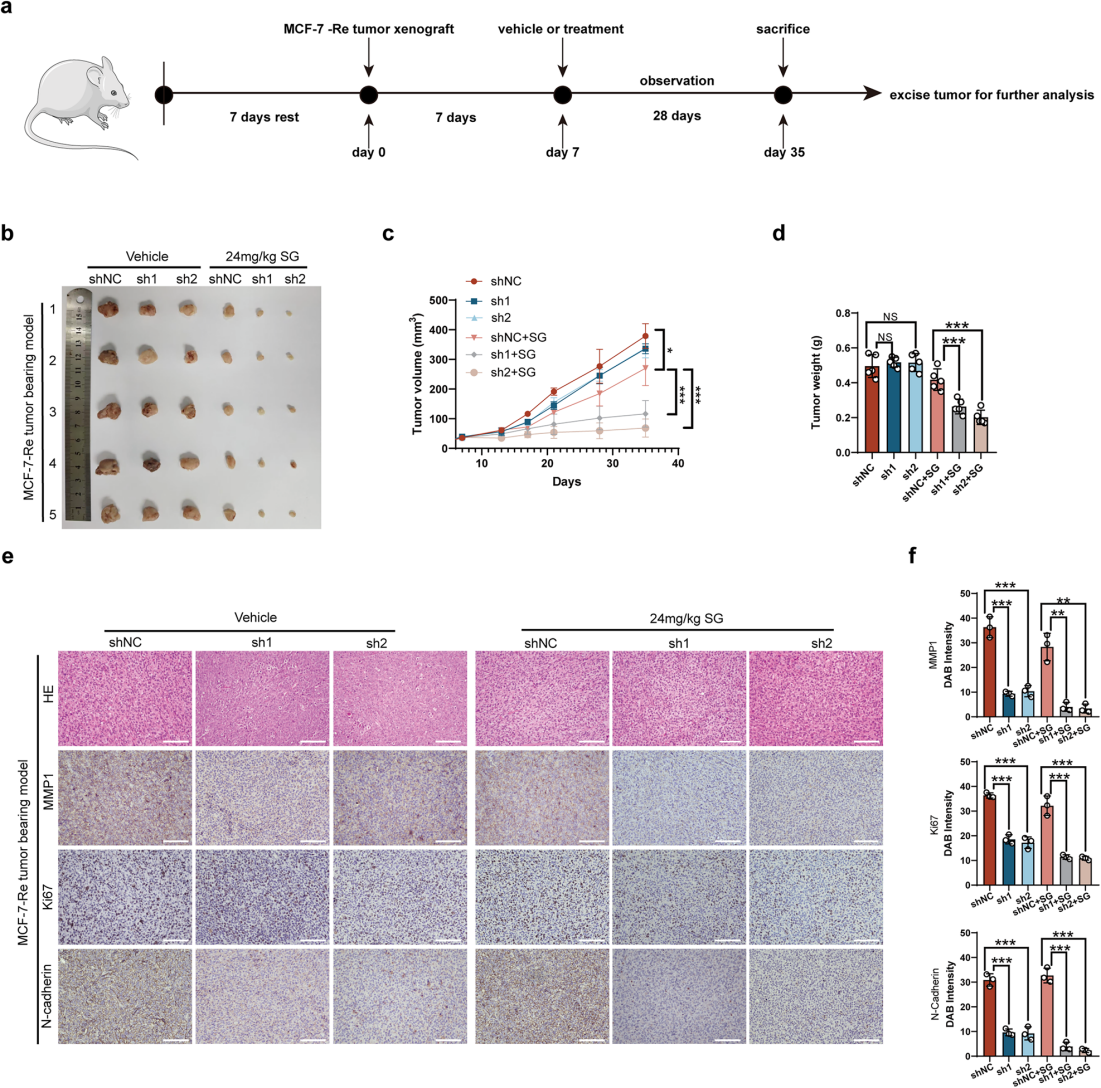
MMP1 inhibition enhances SG sensitivity in SG-resistant BC cells in vivo. **a** Flowchart illustrating the construction of the CDX model with MCF-7 used in this study. **b** Images of harvested tumors from different groups on Day 35. **c** Tumor volume measurements taken every 7 days across different treatment groups (*n* = 5 per group). **d** Tumor weight quantification across different treatment groups (*n* = 5 per group). **e** MMP1 inhibition was significantly associated with decreased expression of Ki67 and N-cadherin, with representative images of different tumors shown in IHC results. **f** Positive rates of Ki67 and N-cadherin. Scale bar: 100 µm. *p*-values were determined using one-way ANOVA. * *p* < 0.05, ** *p* < 0.01, *** *p* < 0.001.

Furthermore, we constructed an animal experimental model as outlined in the flowchart shown in Fig. 7a. MCF-7-Re cells with MMP1-knockdown (shMMP1) or its control (shNC) were subcutaneously inoculated into BALB/c nude mice after a 1-week adaptation period. Each mouse was subsequently injected intraperitoneally with SG (24 mg/kg, every 7 days for 3 weeks). As illustrated in Fig. 7b–d, the tumor sizes and weights in the SG treatment group were smaller than those in the corresponding non-treatment group. Notably, regardless of SG treatment, MMP1 knockdown further enhanced tumor growth inhibition.

Additionally, hematoxylin and eosin (HE) staining, along with IHC staining for Ki67 and EMT-related markers, was conducted on tumor tissue to assess cell proliferation and migration. MMP1 knockdown via shRNA in combination with SG treatment significantly reduced cell proliferation and migration (Fig. 7e, f).

Western blot analysis revealed elevated levels of pIKKα/β, pP65, pIκB, N-cadherin, p-AKT, and vimentin in MMP1-knockdown BC cells compared to the control group (Fig. S3a–c).

### MMP1 inhibition enhances SG-induced cell death

**Fig. 8:**
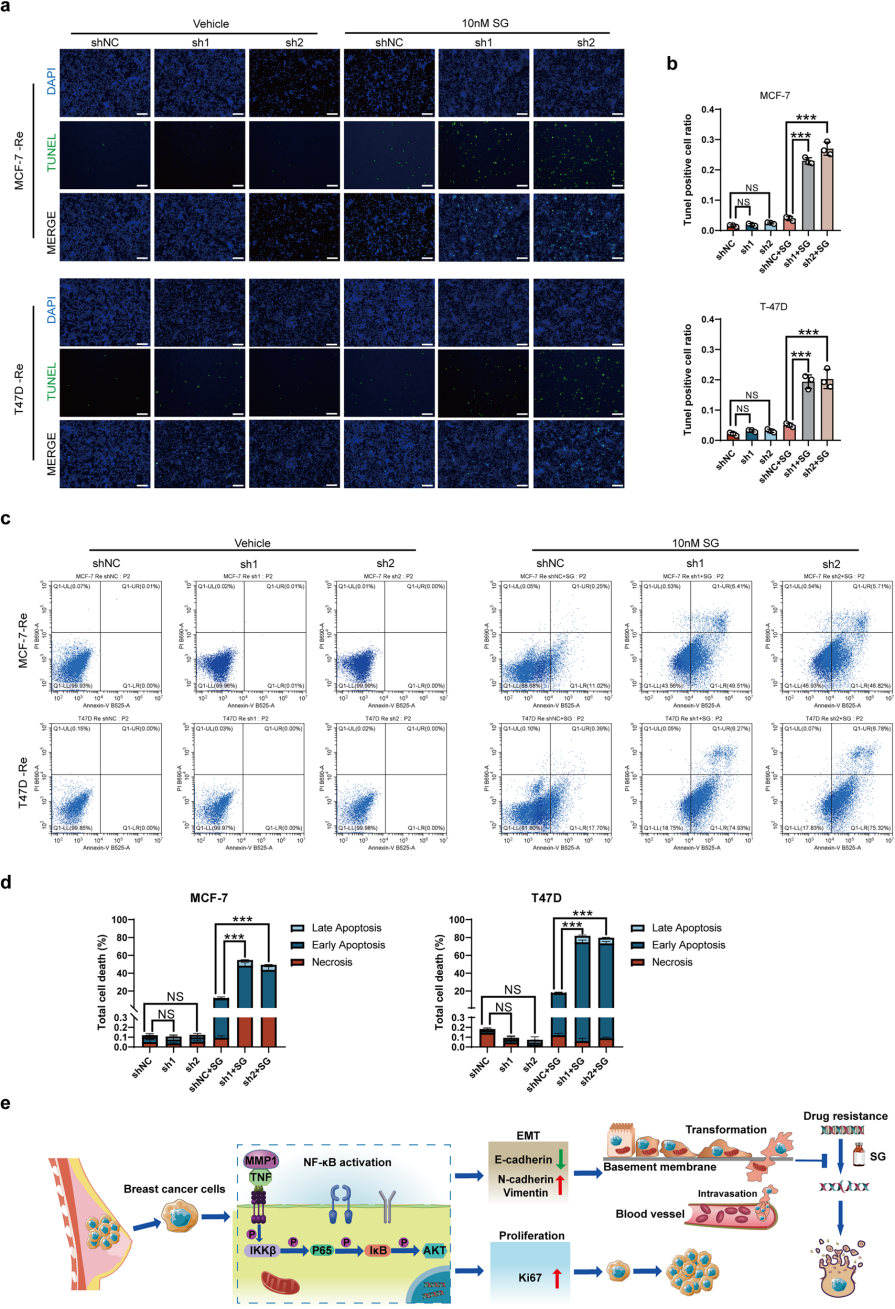
MMP1 inhibition enhances SG-induced cell death. **a**, **b** Comparison of apoptotic cell number between MMP1-knockdown and negative control with or without SG treatment in resistant cell lines via Tunnel assays. **c**, **d** Comparison of apoptotic cell number between MMP1-knockdown and negative control with or without SG treatment in resistant cell lines via Annexin V-PI assays. **e** Proposed mechanisms of MMP1-mediated SG resistance in HR + BC.

In order to preliminarily illustrate the type of cell death of cell lines after treatment of SG, we did Tunnel and Annexin V-PI assays. The rate of cell death significantly increased in MMP1-knockdown combined with SG group compared with control, while without SG, the cell death rate was no difference in merely silencing MMP1 group compared with its control (Fig. 8a, b). In consistence with above result, the Annexin V-PI assay with FACS showed that the apoptosis rate evidently increased in resistant cell lines after combining SG treatment with MMP1 knockdown (Fig. 8c, d).

Therefore, our in vitro and in vivo results indicate that MMP1 inhibition enhanced the efficacy of SG and could represent a promising strategy for reversing SG resistance as shown in the graphical abstract (Fig. 8e).

## Discussion

This study is the first to elucidate the crucial role of MMP1 in SG resistance in BC, demonstrating that the upregulation of MMP1 is closely associated with SG resistance and affects tumor cell migration and invasion through the activation of the NF-κB signaling pathway. Our findings indicate that MMP1 is highly expressed in BC samples, particularly in SG-resistant cells compared to SG-sensitive cells. Analyses from the TCGA database and IHC results suggest that elevated MMP1 expression correlates with poor prognosis in BC patients, including OS and DFS.

Increased MMP1 expression promotes cell proliferation, migration, invasion, and resistance to SG in BC cell lines. Specifically, the inhibition of MMP1 via shRNA significantly enhanced SG sensitivity in MCF-7-Re and T47D-Re cells, while the upregulation of MMP1 conferred SG resistance in MCF-7-Pa and T47D-Pa cells. In vivo experiments further validate MMP1 as an oncogene and an effective therapeutic target in the treatment of HR + BC. The downregulation of MMP1, in conjunction with SG treatment, significantly inhibited tumor growth and migration compared to SG treatment alone, indicating the potential to reverse SG resistance.

Prior research has predominantly focused on the role of MMP1 in tumor invasion and metastasis through ECM degradation [29, 30], with its involvement in chemoresistance being less explored. MMP1 promotes invasion and metastasis partly through the induction of EMT, which also contributes to chemotherapy resistance. MMPs are critical in various biological processes and could serve as biomarkers for diseases such as cancer. However, their varying activities across different substrates and disease stages pose challenges for targeted therapies [31, 32]. In BC therapy, certain MMPs, including MMP1, are promising targets. Given that MMP1-based therapies may lose efficacy as the disease progresses, early inhibition of MMP1 could effectively impede tumor advancement [33].

Previous studies have highlighted the significant role of MMP1 in drug resistance. The miR-145-MMP1 axis has been identified as a regulator of cancer stemness and chemoresistance, making it a promising therapeutic target [34]. MMP1 is also linked to resistance in erlotinib-treated non-small cell lung cancer (NSCLC), where its high expression correlates with poor survival outcomes, suggesting it as a marker of erlotinib resistance [35]. In BC, MMP1 is overexpressed in doxorubicin-resistant cells, and its knockdown sensitizes these cells to the drug. MMP1 contributes to drug resistance through mechanisms such as ECM remodeling, promoting tumor angiogenesis, and activating other resistance-related molecules, which complicates treatment outcomes [36, 37].

In our current study, we demonstrated that MMP1 plays a significant role in chemotherapy resistance by regulating EMT in BC cells. The role of MMP1 is closely associated with the activation of the NF-κB signaling pathway, a critical transcription factor that modulates genes involved in inflammation, immune response, and cell survival. Upon activation, NF-κB translocates to the nucleus, where it binds to the promoter region of the MMP1 gene, initiating its transcription [38]. This mechanism implies that MMP1 is essential for enhancing SG resistance by driving EMT. Our findings are consistent with previous studies indicating that drug-resistant cancer cells are more prone to undergo EMT, thereby highlighting the critical role of MMP1 in this process [24]. Furthermore, our results provide a novel perspective by demonstrating that MMP1 plays a key role in conferring resistance to SG in HR + BC cells through the activation of the NF-κB signaling pathway. We discovered that MMP1 can activate the NF-κB pathway, which subsequently impacts EMT, significantly contributing to SG resistance. This finding not only highlights the importance of MMP1 as a therapeutic target but also offers insights into the underlying mechanisms of drug resistance in BC.

SG-based therapy presents a novel approach for treating advanced BC; however, resistance limits its clinical efficacy. The mechanisms underlying resistance to ADCs include changes in antigen expression, drug internalization, immune evasion, increased drug efflux, enhanced DNA repair, and upregulation of anti-apoptotic factors [39]. EMT also plays a crucial role in tumor metastasis and drug resistance [40, 41]. Previous studies have identified cathepsin L (CTSL)-mediated EMT as a potential target to enhance the efficacy of cisplatin or paclitaxel in lung cancer and other malignancies, revealing that EMT cells exhibit a selective growth advantage in the presence of these drugs. The role of EMT in drug resistance is well-documented across various cancers, including pancreatic cancer [41, 42], bladder cancer [43], and BC [44]. For instance, recent research by Fischer et al. developed an EMT lineage-tracing system in mice, suggesting that EMT cells are more resistant to apoptosis and facilitate metastasis following chemotherapy [45].

However, the specific role of MMP1 in regulating EMT-mediated chemotherapy resistance in BC has not been fully investigated. Our study addresses this gap by revealing that MMP1 significantly contributes to SG resistance in BC cells by regulating EMT. We demonstrated that MMP1 overexpression in SG-sensitive BC cells enhances cell migration and invasion, while its knockdown in SG-resistant cells could reverse these effects. These findings align with previous research indicating that drug-resistant cancer cells are predisposed to undergo EMT, as evidenced by the observed downregulation of E-cadherin and upregulation of N-cadherin and vimentin in SG-resistant BC cells [46].

Furthermore, our study uniquely identifies the NF-κB signaling pathway as a key mediator in this process [47, 48]. The results suggest that MMP1 may serve as a predictive biomarker for SG resistance in HR + BC patients (Fig. S3d). By identifying patients with high MMP1 expression levels, clinicians can optimize treatment strategies more effectively. For instance, combining MMP1 inhibitors with SG therapy could potentially enhance therapeutic efficacy and improve patient outcomes. Future clinical trials should assess the efficacy of MMP1-targeted therapies in conjunction with standard SG treatment protocols. Targeting MMP1 not only opens avenues for enhancing the effectiveness of existing therapies but also holds promise for improving OS rates for HR + BC patients. By reversing SG resistance, patients may achieve better disease control and extended remission periods. Ultimately, this research underscores the significance of personalized medicine, demonstrating that understanding the molecular mechanisms underlying drug resistance can result in more effective and individualized treatment strategies, thereby improving patient care and quality of life.

However, our current study has several limitations, particularly the lack of investigation into the differences in serum MMP1 levels between SG-sensitive and SG-resistant BC patients. Additionally, the experimental design may not fully capture the complexity of the tumor microenvironment in vivo. While in vitro studies provide valuable insights, they may not fully account for the interactions between tumor cells and the surrounding stromal or immune cells, which could influence MMP1 expression and its role in drug resistance. While direct evidence linking SG treatment to MMP1 upregulation is still emerging, there are several potential mechanisms and signaling pathways that could be activated or inhibited in this context. The PI3K/Akt/mTOR and MAPK/ ERK signaling pathway is frequently activated in cancer and has been associated with resistance to chemotherapy and targeted therapies. This pathway regulates key cellular processes such as metabolism, growth, and survival. It is known that PI3K/Akt/mTOR signaling can upregulate MMP1 expression. In the context of SG resistance, activation of this pathway might provide a survival advantage to cancer cells by promoting ECM remodeling, migration, and invasion. The p53 tumor suppressor gene is frequently mutated or inactivated in many cancers, contributing to resistance to chemotherapy. In the case of SG, the release of SN-38 from the ADC is known to induce DNA damage response. A disrupted p53 pathway could lead to the activation of compensatory mechanisms, including the upregulation of MMP1. Additionally, the loss of p53 function might impair the ability of cancer cells to undergo apoptosis, thereby allowing them to survive and proliferate in the presence of SG. A better understanding of how these potential pathways are modulated by SG treatment could provide insights into potential combination strategies that target both MMP1 expression and the underlying signaling networks. Moreover, patient-specific factors such as age, hormonal status, and genetic background could also impact MMP1 expression and its association with SG resistance. These individual variations might lead to differences in treatment responses and outcomes, emphasizing the need for personalized approaches in future research.

To overcome these limitations, future studies should include a larger and more diverse patient population and incorporate longitudinal analyses to monitor MMP1 levels over time in relation to treatment outcomes. Additionally, employing multi-omics approaches could provide deeper insights into the molecular mechanisms underlying MMP1’s role in drug resistance. Exploring the interplay between MMP1 and other signaling pathways, as well as the influence of the tumor microenvironment, will be crucial for developing comprehensive therapeutic strategies. A deeper understanding of how these factors affect the function of MMP1 in various contexts will enhance the efficacy of targeted therapies.

## Conclusion and perspectives

In conclusion, the present study revealed that MMP1 was highly expressed in SG-resistant BC cells and tissues, and its elevated expression was associated with poor prognosis in BC patients. MMP1 has been shown to activate the NF-κB pathway and influence EMT, thereby contributing to SG resistance. Genetic knockdown and pharmacological inhibition of MMP1 restored SG sensitivity both in vitro and in vivo. These findings underscore the potential of targeted inhibition of MMP1 as a strategy to reverse SG resistance and mitigate metastatic properties in BC. Future research should focus on exploring the clinical applicability of MMP1 as a biomarker and therapeutic target within personalized treatment strategies.

## Supplementary information

Original data in article

The supplementary material

## Data Availability

The datasets generated during and/or analyzed during the current study are available from the corresponding author on reasonable request.
