# Supplementary figures and images for: MMP1-induced NF-κB activation promotes epithelial–mesenchymal transition and sacituzumab govitecan resistance in hormone receptor-positive breast cancer
Source: Cell Death Dis. 2025 Apr 26;16(1):346. doi: 10.1038/s41419-025-07615-y (PMC12033297; doi:10.1038/s41419-025-07615-y)

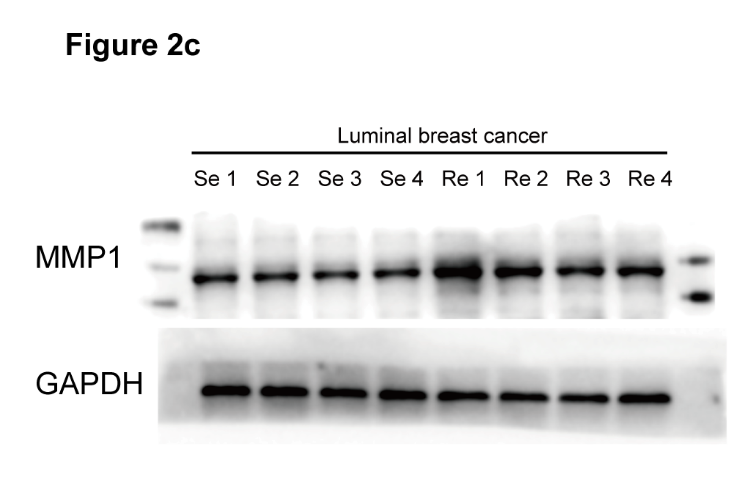

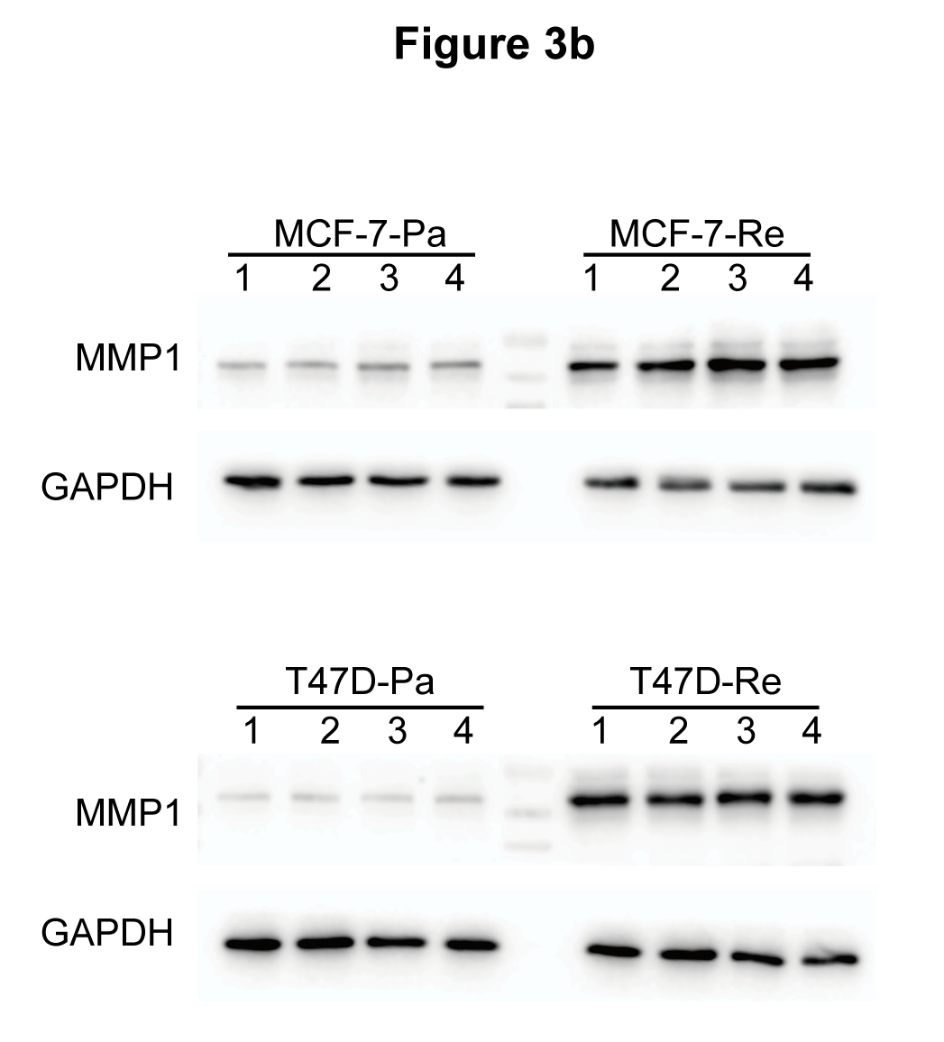

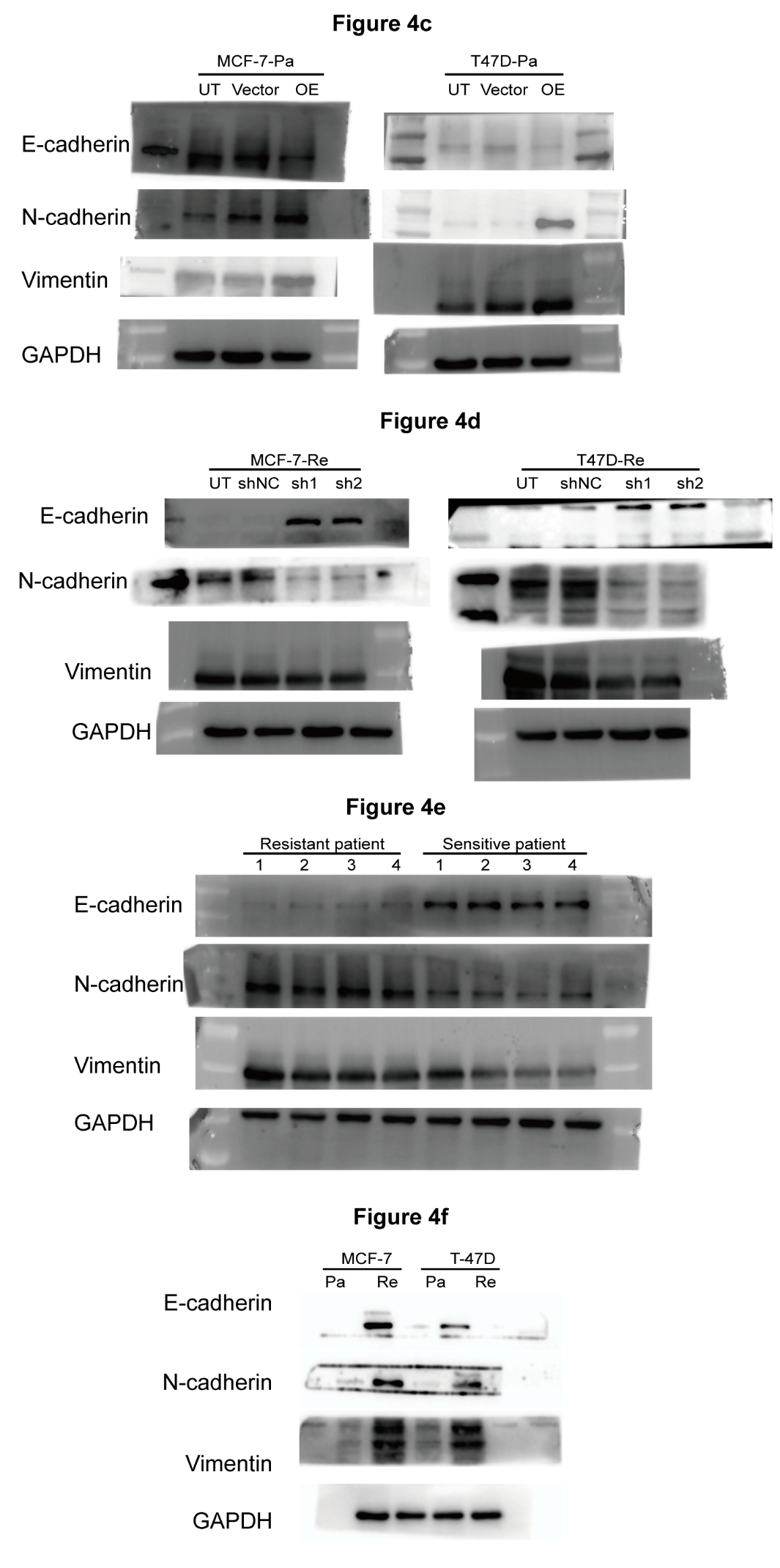

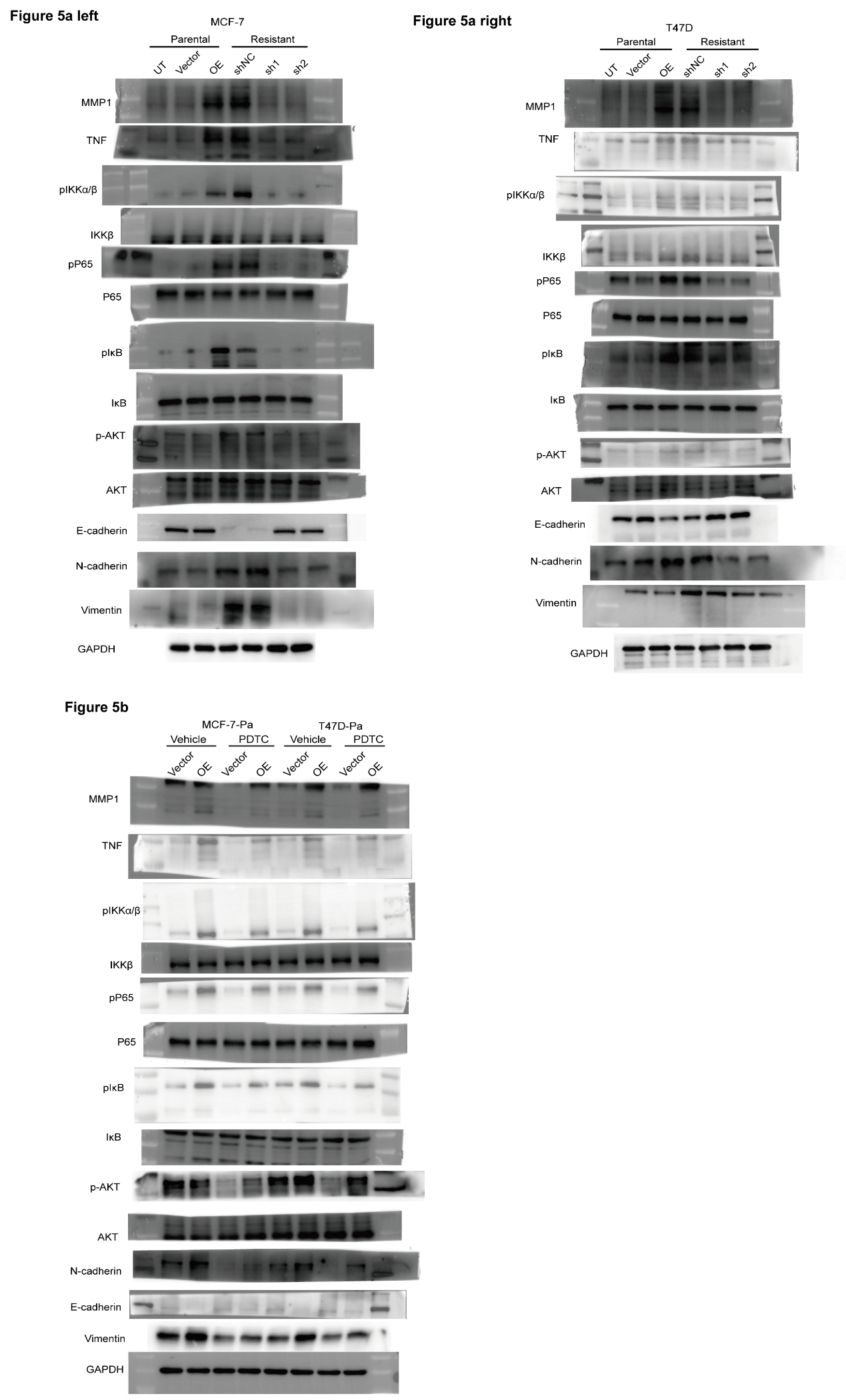

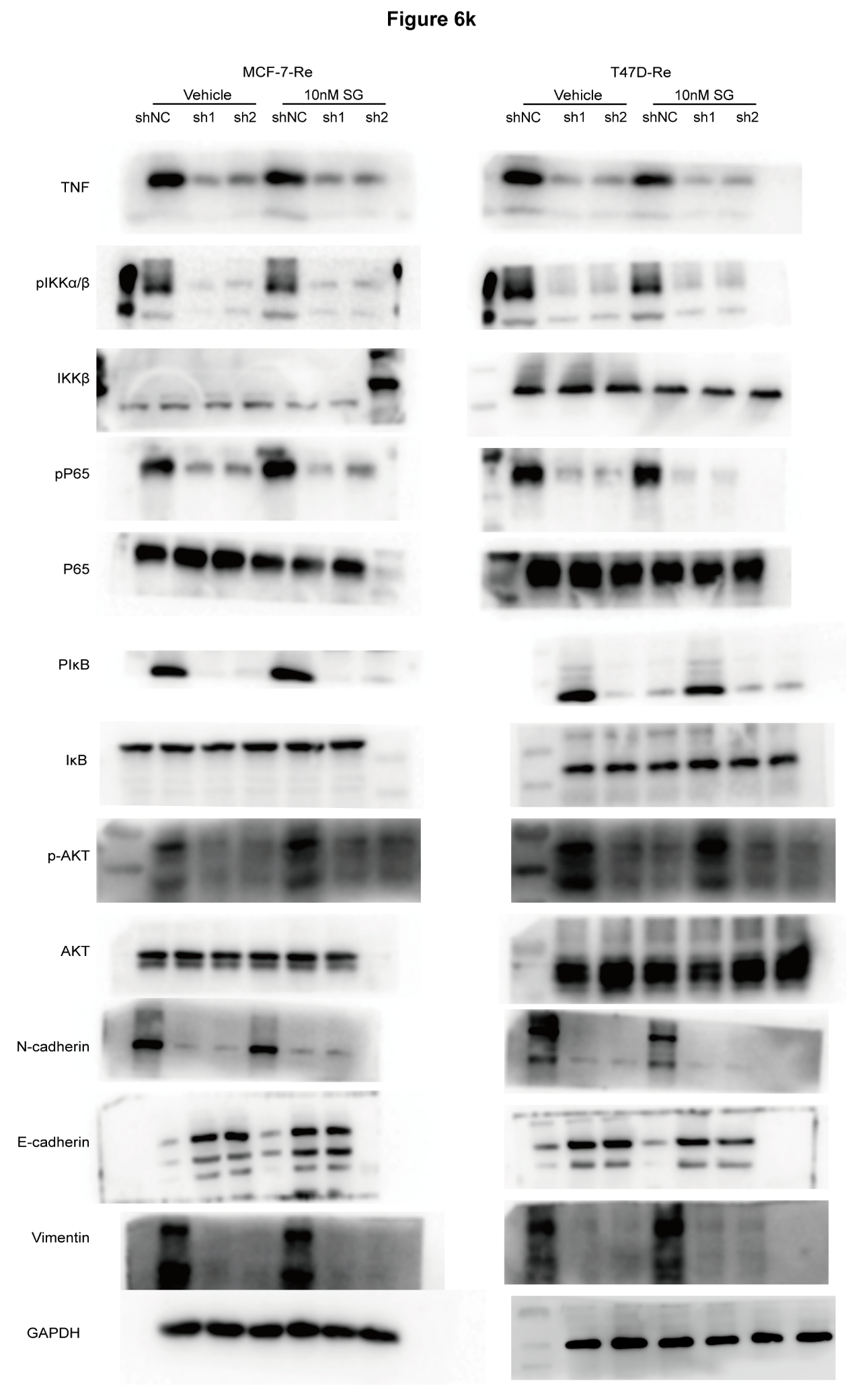

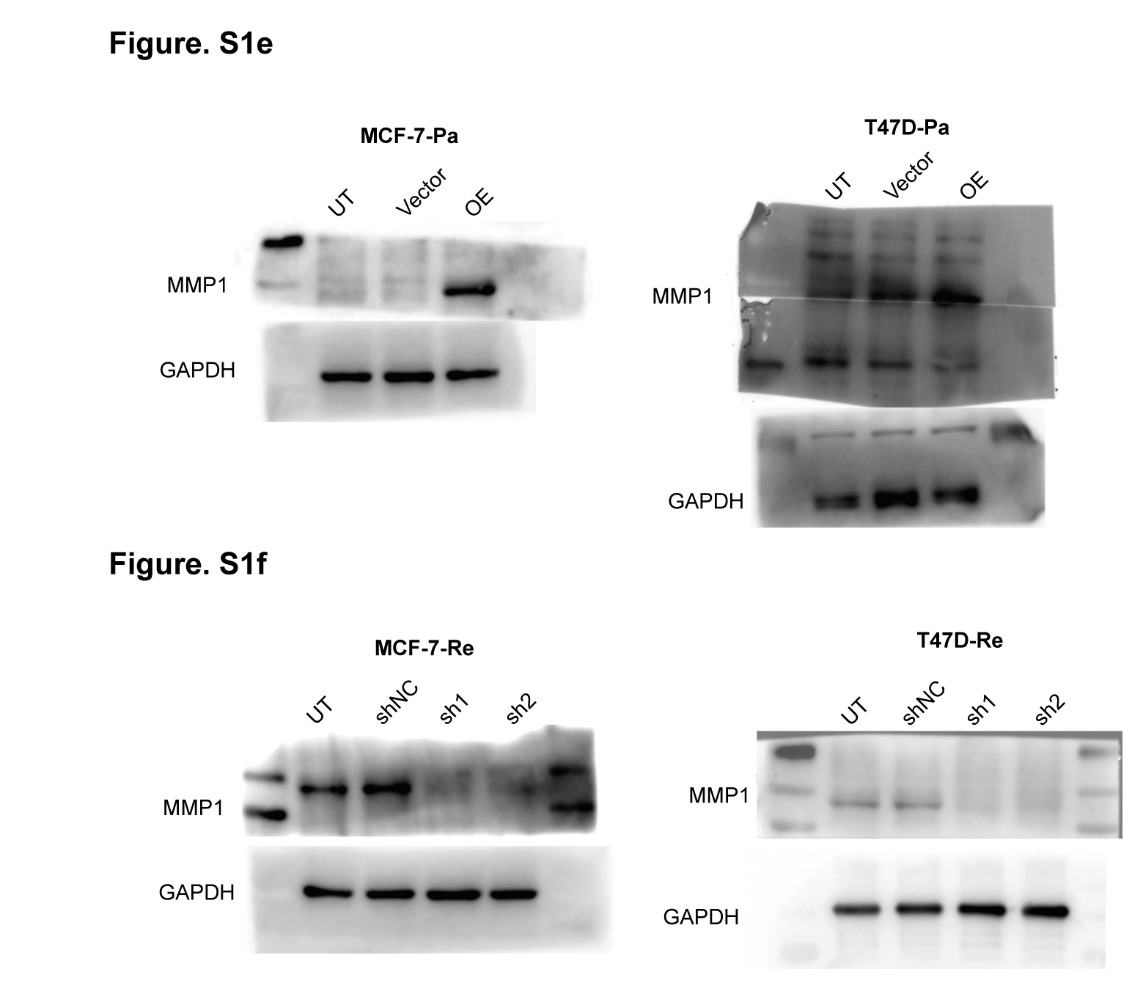


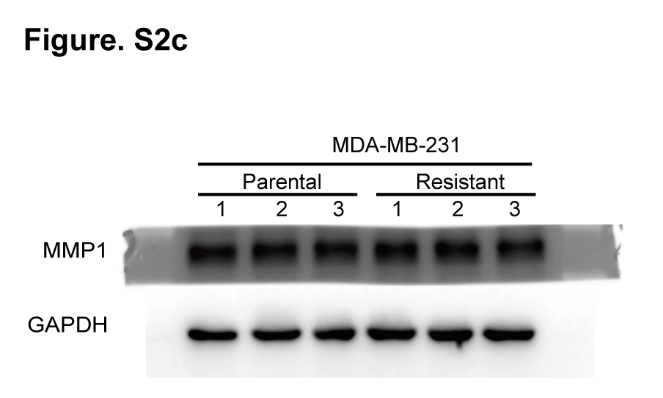


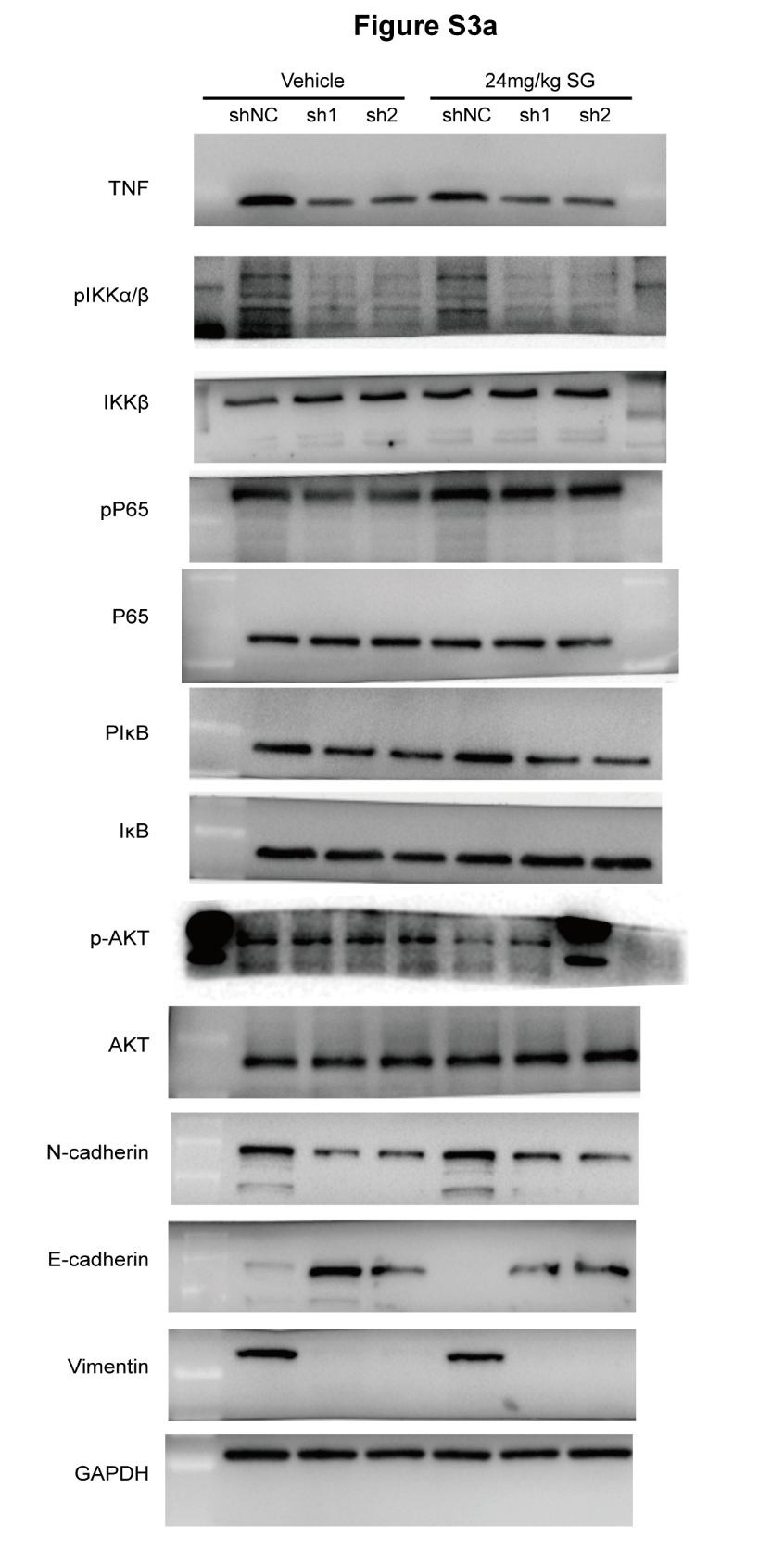

Supplement: Supplementary file 1 — Original data in article [file 41419_2025_7615_MOESM1_ESM.docx]
