# Supplementary material for: MMP1-induced NF-κB activation promotes epithelial–mesenchymal transition and sacituzumab govitecan resistance in hormone receptor-positive breast cancer
Source: Cell Death Dis. 2025 Apr 26;16(1):346. doi: 10.1038/s41419-025-07615-y (PMC12033297; doi:10.1038/s41419-025-07615-y)
Supplement: Supplementary file 2 — The supplementary material [file 41419_2025_7615_MOESM2_ESM.pdf]

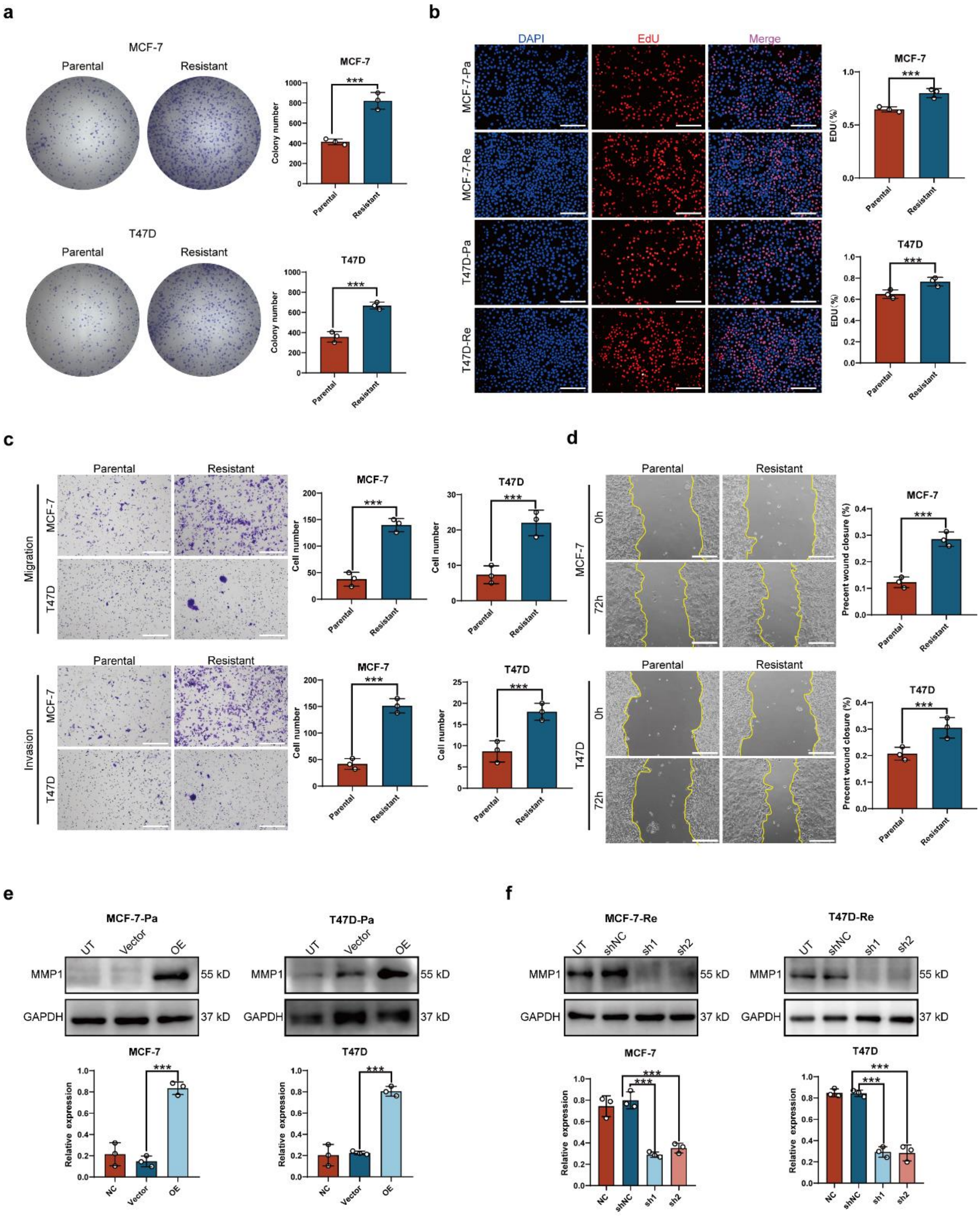
a
MCF-7
Parental
Resistant
MCF-7
Colony number
***
Parental
Resistant
T47D
Parental
Resistant
T47D
Colony number
***
Parental
Resistant
b
DAPI
EdU
Merge
MCF-7-Pa
MCF-7-Re
T47D-Pa
T47D-Re
MCF-7
EDU(%)
***
Parental
Resistant
T47D
EDU(%)
***
Parental
Resistant
c
Parental
Resistant
Migration
MCF-7
T47D
MCF-7
Cell number
***
Parental
Resistant
T47D
Cell number
***
Parental
Resistant
Parental
Resistant
Invasion
MCF-7
T47D
MCF-7
Cell number
***
Parental
Resistant
T47D
Cell number
***
Parental
Resistant
d
Parental
Resistant
MCF-7
0h
72h
MCF-7
Precent wound closure (%)
***
Parental
Resistant
Parental
Resistant
T47D
0h
72h
T47D
Precent wound closure (%)
***
Parental
Resistant
e
MCF-7-Pa
UT
Vector
OE
MMP1
55 kD
GAPDH
37 kD
T47D-Pa
UT
Vector
OE
MMP1
55 kD
GAPDH
37 kD
MCF-7
Relative expression
***
NC
Vector
OE
T47D
Relative expression
***
NC
Vector
OE
f
MCF-7-Re
UT
shNC
sh1
sh2
MMP1
55 kD
GAPDH
37 kD
T47D-Re
UT
shNC
sh1
sh2
MMP1
55 kD
GAPDH
37 kD
MCF-7
Relative expression
***
***
NC
shNC
sh1
sh2
T47D
Relative expression
***
***
NC
shNC
sh1
sh2

Fig. S1. Characterization of SG-sensitive and resistant BC cell lines.

a Comparison of cell proliferation via colony formation assays in SG-sensitive and SG-resistant cell lines. **b** EdU assays were conducted to assess the proliferation ability between the two groups. **c** Transwell migration and invasion assays comparing SG-sensitive versus SG-resistant cells. **d** Wound scratch assays were employed to measure cell migration in both SG-sensitive and SG-resistant cells. **e** Western blot analysis confirmed MMP1 overexpression in MCF-7-Pa and T47D-Pa cells. **f** Western blot analysis confirmed MMP1 silencing in MCF-7-Re and T47D-Re cells. GAPDH served as an internal control. Scale bar: 100 μm. *p*-values were determined using a two-tailed unpaired Student's t-test. ** $p < 0.01$, *** $p < 0.001$, ns: non-significance.

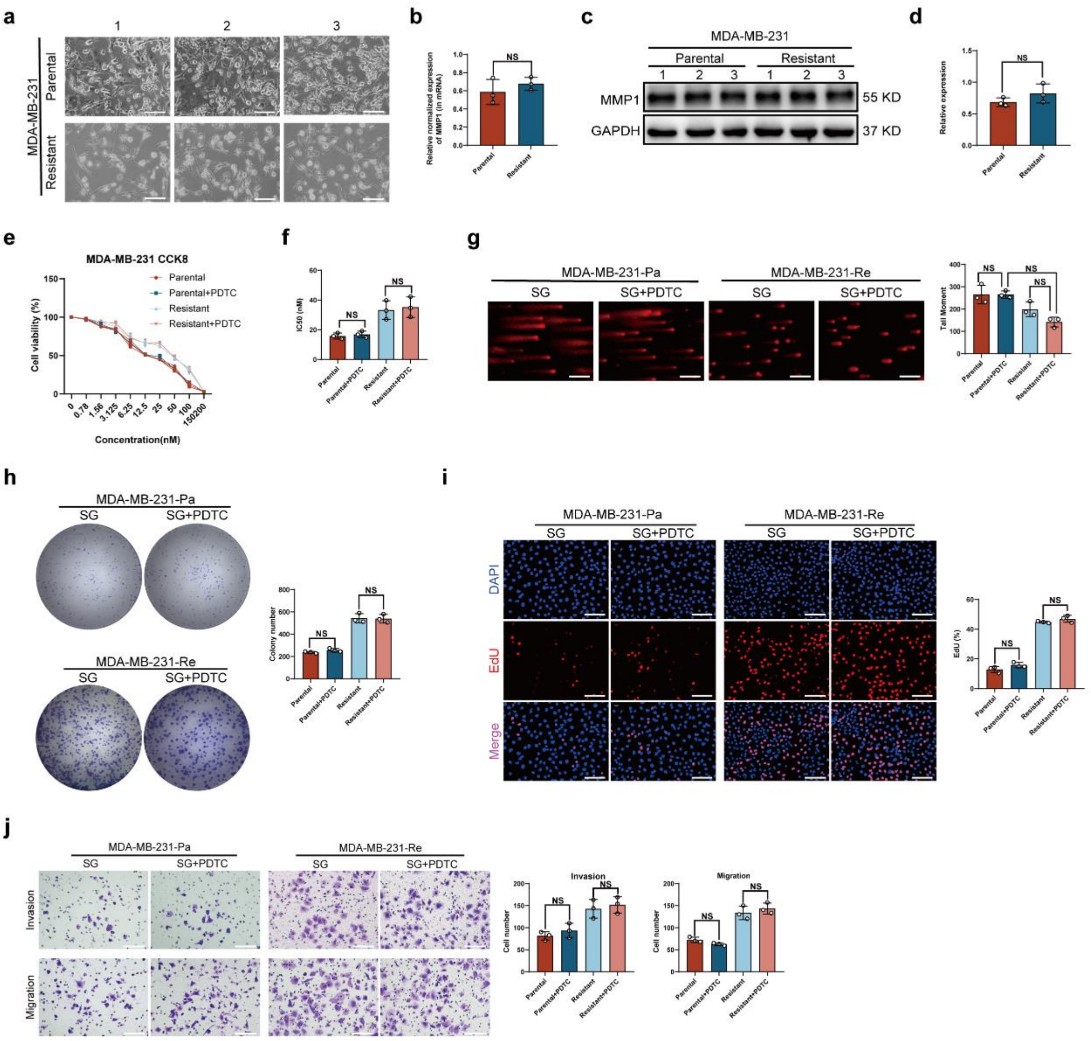
a
1
2
3
MDA-MB-231
Parental
Resistant
b
NS
Relative normalized expression of MMP1 (in mRNA)
Parental
Resistant
c
MDA-MB-231
Parental
Resistant
MMP1
55 KD
GAPDH
37 KD
d
NS
Relative expression
Parental
Resistant
e
MDA-MB-231 CCK8
Parental
Parental+PDTC
Resistant
Resistant+PDTC
Cell viability (%)
Concentration(nM)
f
NS
IC50 (nM)
Parental
Parental+PDTC
Resistant
Resistant+PDTC
g
MDA-MB-231-Pa
MDA-MB-231-Re
SG
SG+PDTC
Tail Moment
h
MDA-MB-231-Pa
MDA-MB-231-Re
SG
SG+PDTC
Colony number
i
MDA-MB-231-Pa
MDA-MB-231-Re
SG
SG+PDTC
DAPI
EdU
Merge
EdU (%)
j
MDA-MB-231-Pa
MDA-MB-231-Re
SG
SG+PDTC
Invasion
Migration
Cell number

Fig. S2. Silencing MMP1 in MDA-MB-231 cell line did not affect the EMT and NF-κB pathway in the CDX model.

a Cell morphology was observed under a microscope. **b** qRT-PCR results showing MMP1 mRNA levels in three BC cell lines and their corresponding resistant cell lines with different molecular subtypes. **c, d** Western blot analysis measuring MMP1 protein levels in three BC cell lines and their corresponding resistant cell lines with different molecular subtypes. **e, f** After knockdown or overexpression of MMP1, the viability of MDA-MB-231-Re was tested at different concentrations of SG using CCK-8 assays. IC50 values for all groups were calculated using GraphPad and compared using Student's t-test. Scale bar: 100 μm. **g** DNA damage assessed by comet assays and quantification of tail moments in MDA-MB-231-Pa and -Re cells treated with 10 nM SG with/without PDTC for 24 hours. **h** Representative images of colony formation assays from MDA-MB-231-Pa and MDA-MB-231-Re treated with MMP1 shRNA or its control for 48 hours, followed by treatment with 10 nM SG for 24 hours. **i** Representative images of EdU assays from MDA-MB-231-Pa and MDA-MB-231-Re treated with MMP1 shRNA or its control for 48 hours, followed by treatment with 10 nM SG for 24 hours. **j** Representative images of transwell assays assessing invasion and migration ability in MDA-MB-231-Pa and MDA-MB-231-Re treated with MMP1 shRNA or its control for 48 hours, followed by treatment with 10 nM SG for 24 hours.

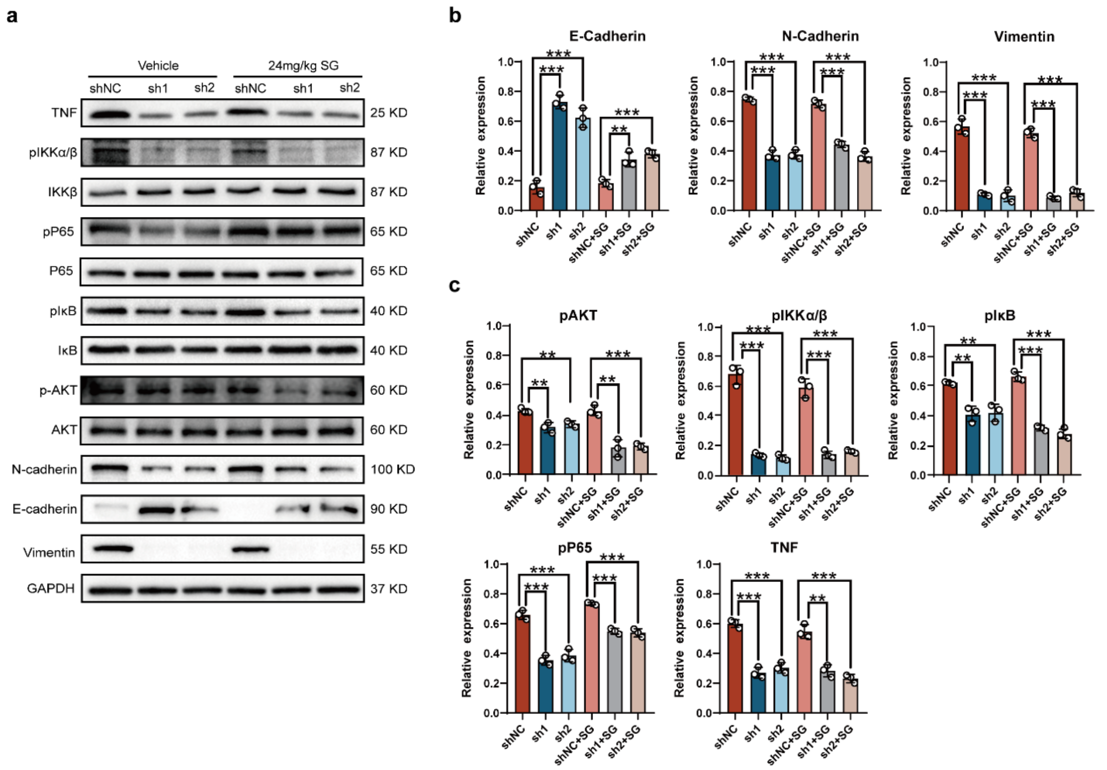

Fig. S3. Silencing MMP1 suppressed EMT and NF-κB pathway in the CDX model.

a Expression levels of MMP1, TNF, pIKKα/β, IKKβ, pP65, P65, pIκB, IκB, AKT, and p-AKT, and different EMT proteins (N-cadherin, E-cadherin, and vimentin) were measured by Western blot. **b, c** Quantitative analysis of panel a by Western blot is presented in histogram form. GAPDH served as an internal control. Data are from three independent experiments. * $p < 0.05$, ** $p < 0.01$, *** $p < 0.001$. **d** Model depicting the MMP1/NF-κB/AKT axis inducing SG resistance via EMT.

Table S1. Details of all differential expression genes

Symbol	log2(fc)	p Value	q Value
BCL2A1	12.17461	4.77E-19	3.97E-18
COMMD3-BMI1	11.57033	9.53E-10	4.24E-09
TM4SF19-	10.62845	3.03E-08	1.18E-07
WFDC3	10.56796	7.69E-06	2.37E-05
PI3	10.55938	0.000123	0.000328
ZNF177	10.07682	7.54E-12	3.93E-11
TP53TG3D	9.900867	1.93E-06	6.30E-06
COL20A1	9.280771	1.20E-10	5.72E-10
CLDN5	9.154818	2.41E-07	8.63E-07
CPEB1	9.092757	0.000979	0.002299
BLOC1S5-	8.985842	3.03E-08	1.18E-07
EPB41L3	8.888743	0.000979	0.002299
PCDHGB3	8.370687	9.64E-07	3.24E-06
MAT1A	8.049849	6.14E-05	0.00017
PKHD1	7.383704	2.38E-13	1.37E-12
SGCZ	6.584963	0.00049	0.001199
SERPINB2	5.344062	4.36E-59	1.75E-57
EDN2	5.283454	4.77E-10	2.17E-09
HMSD	5.053626	1.41E-07	5.15E-07
CDYL2	4.826272	4.52E-52	1.39E-50
KCNK6	4.807355	4.82E-07	1.68E-06
MMP1	4.653247	1.04E-11	5.40E-11
CSMD3	4.629357	3.85E-06	1.22E-05
SLAMF8	4.598259	0.00049	0.001199
MYO7B	4.464108	4.82E-07	1.68E-06
RTBDN	4.451642	4.04E-28	5.28E-27
PTPN22	4.295393	2.74E-66	1.31E-64
PCDHB7	4.203442	2.46E-16	1.75E-15
TRIM29	3.916672	0.000122	0.000327
CGB8	3.891957	1.14E-07	4.20E-07
F2RL3	3.678739	1.04E-46	2.70E-45
GPR87	3.627703	1.34E-07	4.90E-07
H4C14	3.506118	3.96E-11	1.97E-10
ABI3BP	3.461487	2.74E-26	3.29E-25
CCL20	3.436138	8.31E-14	4.92E-13
CYP4F11	3.420957	4.04E-05	0.000114
SFTPB	3.246214	5.28E-09	2.21E-08
CCR10	3.237603	7.81E-07	2.65E-06
TREM1	3.23349	9.41E-14	5.57E-13
ZNF836	3.225823	0.000223	0.000576
PCDHB4	3.144046	0.000521	0.001271
RELN	3.093976	3.62E-05	0.000103
LRTOMT	3.060542	0.000732	0.001755
TMEM45A	3.047461	2.73E-78	1.67E-76
CXCL3	3.013014	1.45E-15	9.69E-15
CXCL1	3.011733	2.08E-76	1.24E-74
HOXB4	2.997493	0.000122	0.000327

TSTD3	2.962996	0.000829	0.001969
MYO16	2.922658	6.68E-15	4.25E-14
SPANXD	2.913439	6.63E-47	1.73E-45
PODXL2	2.905649	1.15E-16	8.33E-16
DHRS2	2.879375	2.31E-78	1.42E-76
SERPINB5	2.857981	1.11E-06	3.70E-06
MEI1	2.83917	8.94E-06	2.72E-05
GRID1	2.807355	6.17E-07	2.12E-06
PKDREJ	2.800691	1.11E-05	3.35E-05
IL13RA2	2.787757	1.11E-05	3.35E-05
GSG1	2.756345	2.23E-11	1.12E-10
SLC16A12	2.732304	0.000732	0.001755
TPTEP2-CSNK1E	2.727671	0.000158	0.000418
ISM1	2.723861	1.11E-06	3.70E-06
KIAA0319	2.720477	6.17E-07	2.12E-06
SPNS2	2.64689	0.000279	0.000711
ADAMTS13	2.638957	0.000185	0.000483
CT83	2.598241	1.93E-19	1.64E-18
NCKAP1L	2.589527	7.88E-38	1.55E-36
HLA-DPA1	2.580076	1.20E-06	3.99E-06
ADTRP	2.560715	4.04E-05	0.000114
GRB14	2.546265	1.64E-08	6.52E-08
PDCD6-AHRR	2.519293	8.88E-19	7.31E-18
TUBAL3	2.491303	8.51E-10	3.80E-09
PCDHB8	2.482883	9.62E-22	9.13E-21
CSF2	2.454402	2.10E-26	2.53E-25
MKKS	2.454295	2.99E-15	1.95E-14
COL17A1	2.439343	3.71E-17	2.75E-16
SCN5A	2.436099	4.38E-29	6.04E-28
PTGS2	2.421017	7.80E-59	3.09E-57
PCDHB16	2.417176	2.78E-33	4.51E-32
SCGB2B2	2.403356	2.50E-09	1.08E-08
OAS2	2.399949	2.41E-27	3.04E-26
RNF43	2.377602	0.000114	0.000307
ALOXE3	2.374808	7.08E-07	2.41E-06
LY6G5C	2.365331	0.000861	0.002038
PTPRR	2.357552	2.27E-05	6.64E-05
TMEM45B	2.355659	1.10E-20	9.95E-20
GNAL	2.334537	9.85E-84	6.82E-82
LRRC24	2.331435	0.000861	0.002038
LDOC1	2.317743	5.02E-80	3.22E-78
CFAP251	2.313635	5.85E-28	7.60E-27
AZGP1	2.291792	2.02E-06	6.58E-06
SYTL2	2.291649	5.88E-07	2.03E-06
CALCRL	2.287802	3.14E-09	1.34E-08
JAK3	2.27739	2.26E-08	8.89E-08
PCDHGC5	2.263034	0.00098	0.002302
PCSK4	2.261265	0.000436	0.001079
SLC12A5	2.243188	1.24E-08	4.98E-08
AOC3	2.205093	3.54E-26	4.22E-25

BRME1	2.202296	8.91E-09	3.64E-08
CFAP70	2.163174	4.13E-05	0.000117
CHURC1-FNTB	2.157421	0.000551	0.00134
GCSAM	2.147237	2.87E-07	1.02E-06
MAPK11	2.130809	5.62E-17	4.12E-16
DCHS1	2.124258	9.91E-20	8.54E-19
GSTM1	2.118	2.46E-29	3.42E-28
IL1B	2.112149	6.64E-78	4.02E-76
SERPING1	2.109952	2.21E-10	1.03E-09
NUDT7	2.101538	2.10E-06	6.83E-06
PAX8	2.095639	1.51E-09	6.61E-09
RTL9	2.072044	9.13E-09	3.72E-08
NUTM2G	2.071553	0.000886	0.002095
C3	2.062657	1.06E-15	7.17E-15
PRSS3	2.046074	1.11E-25	1.29E-24
ANKLE1	2.041619	7.52E-07	2.56E-06
C1orf116	2.040642	4.27E-05	0.00012
RNF39	2.037839	2.54E-05	7.39E-05
MAP7	2.026954	1.56E-27	1.99E-26
SHC3	2.025786	3.71E-55	1.29E-53
P2RY11	2.022616	3.81E-41	8.40E-40
PMEL	2.019754	1.08E-06	3.63E-06
COL8A1	2.017628	1.34E-53	4.40E-52
TSPAN7	1.995573	4.68E-07	1.63E-06
SPANXA2	1.995046	1.50E-50	4.47E-49
C6orf226	1.992806	6.56E-08	2.48E-07
KRTAP2-3	1.992786	5.97E-93	4.84E-91
MAP3K7CL	1.98871	7.52E-07	2.56E-06
PCDHB13	1.98806	2.44E-08	9.60E-08
PDGFB	1.974506	3.70E-12	1.97E-11
PCDHB10	1.957245	8.32E-13	4.64E-12
EDN1	1.94627	5.55E-30	7.95E-29
KCNK3	1.942179	0.000316	0.000798
B3GNT4	1.941514	2.04E-06	6.64E-06
ZGLP1	1.93698	4.30E-05	0.000121
CHKB-CPT1B	1.93584	8.35E-23	8.40E-22
CYP3A5	1.926987	2.52E-15	1.66E-14
C6orf141	1.920687	6.09E-20	5.27E-19
DCT	1.91908	4.13E-05	0.000117
DNAH3	1.916806	3.29E-06	1.05E-05
CFAP58	1.892085	9.38E-05	0.000254
CXCL2	1.889452	1.52E-19	1.30E-18
NFKBIZ	1.889027	1.10E-67	5.45E-66
TESPA1	1.877488	4.22E-23	4.31E-22
MEGF6	1.852786	1.20E-42	2.76E-41
KCNMA1	1.851749	0.000122	0.000327
ST6GALNAC5	1.846605	1.51E-10	7.16E-10
PRRG4	1.84405	2.30E-05	6.72E-05
PRR22	1.841435	4.70E-13	2.67E-12
ZNF165	1.839535	0.000478	0.001173

PRICKLE4	1.836779	0.000275	0.000702
ARHGAP24	1.82738	2.43E-21	2.26E-20
PLEKHB1	1.820086	9.06E-15	5.70E-14
ADAMTS6	1.813149	3.81E-13	2.17E-12
HOXC8	1.807929	1.93E-11	9.75E-11
VAV1	1.80516	3.97E-34	6.68E-33
IFI27L2	1.801717	4.18E-25	4.74E-24
MYH15	1.794955	2.63E-09	1.13E-08
PSPN	1.785277	8.73E-14	5.16E-13
CORO2B	1.784159	5.30E-40	1.13E-38
PAEP	1.773668	1.09E-23	1.14E-22
MEF2B	1.761292	1.66E-10	7.83E-10
LNP1	1.752907	1.31E-07	4.81E-07
FAM86B1	1.751605	1.03E-08	4.17E-08
EIF3CL	1.750771	1.24E-09	5.47E-09
MCF2L2	1.744743	0.000631	0.001523
FAM24B	1.740721	1.51E-18	1.23E-17
BORCS8-MEF2B	1.740538	5.19E-18	4.10E-17
COQ5	1.738893	1.49E-50	4.43E-49
AOC2	1.738523	2.17E-33	3.52E-32
DNAJC12	1.734913	4.57E-14	2.76E-13
PCDHB2	1.734761	3.26E-09	1.39E-08
CFAP20DC	1.732202	1.89E-07	6.81E-07
ARG2	1.729833	2.78E-20	2.47E-19
MAST4	1.719251	9.26E-53	2.90E-51
ADAMTS1	1.717027	3.93E-05	0.000111
UNC5CL	1.705887	0.000614	0.001485
LURAP1L	1.705604	2.57E-10	1.19E-09
EMILIN1	1.70543	0.000182	0.000476
PPP1R3E	1.704779	8.81E-23	8.85E-22
H1-2	1.703854	4.01E-46	1.02E-44
SRPX2	1.699491	0.000191	0.000498
LNX1	1.684498	1.06E-09	4.68E-09
PRR29	1.683919	3.41E-06	1.09E-05
ADM5	1.681262	0.000952	0.00224
SLC27A3	1.679927	1.40E-14	8.72E-14
PROS1	1.676939	5.02E-99	4.54E-97
CTXN1	1.672987	1.92E-15	1.27E-14
MAP1A	1.671471	1.18E-09	5.19E-09
PCDHB15	1.65983	7.50E-05	0.000206
STK32A	1.655736	1.85E-06	6.07E-06
EML1	1.652115	8.91E-18	6.91E-17
PDE2A	1.651587	1.29E-28	1.73E-27
APCDD1L	1.648738	3.42E-05	9.80E-05
PCDHB14	1.63743	2.44E-08	9.60E-08
ZCWPW1	1.636582	4.37E-07	1.53E-06
SMIM14	1.631805	3.10E-26	3.70E-25
C17orf113	1.628906	0.000202	0.000525
GFAP	1.625093	1.30E-21	1.22E-20
FBXL2	1.624331	4.57E-27	5.67E-26

ARMC5	1.621975	9.60E-29	1.30E-27
CALHM5	1.61471	9.70E-07	3.26E-06
FAM53A	1.601876	0.000134	0.000357
WDR25	1.598893	1.03E-22	1.03E-21
C12orf76	1.589169	2.16E-09	9.36E-09
ODAD3	1.58742	4.82E-05	0.000135
MLLT11	1.583253	9.03E-13	5.01E-12
PCDHB9	1.549991	2.86E-10	1.32E-09
DUSP19	1.549816	8.93E-06	2.72E-05
CLDN15	1.53905	2.29E-38	4.61E-37
ICAM2	1.536307	8.24E-41	1.80E-39
TNFAIP3	1.534794	1.88E-58	7.33E-57
IFFO1	1.532146	0.000362	0.000906
ATP6V1E2	1.523412	1.01E-07	3.75E-07
ZNF503	1.522555	3.27E-27	4.09E-26
ZFP41	1.503273	1.79E-54	6.12E-53
HSD11B1L	1.500786	0.000154	0.000407
PER3	1.498578	2.11E-12	1.14E-11
TRANK1	1.498487	8.58E-28	1.10E-26
VSIG10L	1.493282	3.03E-05	8.72E-05
CFAP410	1.490601	6.62E-21	6.02E-20
KLF7	1.490132	3.30E-53	1.06E-51
CTF1	1.48434	1.10E-10	5.27E-10
KDM4D	1.469821	3.02E-06	9.69E-06
RFXAP	1.468006	5.26E-07	1.82E-06
LTBP2	1.466	3.28E-14	2.00E-13
PLEK2	1.453351	4.10E-83	2.76E-81
SCN2A	1.451815	1.61E-22	1.59E-21
TMEM225B	1.44489	7.65E-05	0.00021
FGFBP1	1.442804	0.000344	0.000864
GPR39	1.437914	2.00E-14	1.23E-13
PCDHB11	1.431674	1.45E-05	4.35E-05
APLP1	1.428903	1.82E-36	3.36E-35
PKIB	1.428282	6.58E-09	2.73E-08
DNAJB2	1.423604	2.25E-53	7.30E-52
SYT17	1.423571	5.55E-09	2.32E-08
PHF7	1.422624	1.48E-06	4.89E-06
AMH	1.420045	5.12E-10	2.32E-09
IL11RA	1.409829	3.86E-05	0.00011
MAPK8IP2	1.409692	0.000507	0.001238
CCDC69	1.407945	7.95E-48	2.16E-46
ASIC3	1.407241	3.61E-05	0.000103
TBXAS1	1.403274	2.44E-05	7.10E-05
CNBD2	1.402946	0.000313	0.00079
AKR1C3	1.396521	5.10E-31	7.59E-30
ZDHHC23	1.396074	1.18E-25	1.37E-24
TMEM276	1.394511	1.38E-08	5.55E-08
NUTM2A	1.38203	3.94E-08	1.52E-07
ACSM3	1.381521	5.82E-07	2.01E-06
PRRT3	1.378512	0.00017	0.000448

KIFC2	1.374638	1.58E-37	3.05E-36
SPTLC3	1.371456	1.27E-22	1.27E-21
CPT1B	1.368799	3.10E-13	1.78E-12
DCLK2	1.368438	9.19E-09	3.75E-08
EDNRA	1.365166	3.36E-06	1.07E-05
SOD3	1.365127	1.51E-15	1.01E-14
DOCK4	1.363407	1.09E-54	3.74E-53
PARP16	1.357692	8.42E-29	1.15E-27
RAPGEF4	1.351985	6.72E-05	0.000185
C11orf71	1.351788	6.18E-05	0.000171
ID4	1.350406	0.000181	0.000474
NFKBIL1	1.346514	4.47E-25	5.04E-24
EIF4ENIF1	1.344676	5.58E-05	0.000155
ZNF517	1.344088	5.31E-06	1.66E-05
PCDH1	1.342924	4.55E-06	1.43E-05
SYT1	1.342156	4.18E-07	1.46E-06
ZNF501	1.337765	1.44E-05	4.32E-05
MTSS1	1.33479	5.92E-06	1.84E-05
NUTM2D	1.333665	2.79E-12	1.50E-11
ABLIM3	1.331473	6.80E-92	5.35E-90
TCTA	1.330187	1.72E-33	2.81E-32
AKR1C2	1.327266	6.55E-12	3.43E-11
PLXDC2	1.324435	1.88E-06	6.15E-06
ZNF497	1.323988	2.30E-05	6.72E-05
PRR36	1.320016	1.44E-06	4.77E-06
TMEM86A	1.318468	0.000263	0.000673
FGF5	1.314821	8.15E-34	1.35E-32
NRP2	1.30952	6.24E-19	5.16E-18
MRPL40	1.303466	8.00E-37	1.51E-35
ABCC9	1.303342	1.05E-05	3.17E-05
SLC24A1	1.299234	2.36E-17	1.77E-16
FGF1	1.295183	3.72E-06	1.18E-05
WNK4	1.293154	5.74E-06	1.79E-05
FRAT1	1.291995	4.83E-06	1.52E-05
ASPSCR1	1.290833	4.44E-43	1.03E-41
NPDC1	1.29057	5.30E-35	9.18E-34
ADAT3	1.284818	7.42E-14	4.40E-13
ZBTB20	1.283121	1.08E-30	1.60E-29
SH2D3A	1.277579	5.40E-18	4.26E-17
ANGEL1	1.276421	4.54E-15	2.93E-14
MYD88	1.275233	5.21E-47	1.38E-45
MAGI2	1.273648	0.000915	0.002158
ZC2HC1C	1.273331	0.00063	0.00152
SEMA3A	1.270541	1.76E-19	1.50E-18
SULT1C2	1.270158	1.99E-11	1.01E-10
CPED1	1.269607	3.37E-09	1.44E-08
PRRT1	1.266601	0.000263	0.000673
CASP1	1.266554	0.000135	0.000358
DHX58	1.265423	6.51E-08	2.47E-07
OGFOD2	1.258876	1.03E-23	1.09E-22

ARSG	1.255446	6.59E-06	2.04E-05
TGFB1I1	1.254518	1.48E-43	3.48E-42
FZD8	1.251761	4.31E-12	2.28E-11
SMDT1	1.249914	1.22E-15	8.19E-15
ZNF385B	1.249246	2.01E-23	2.09E-22
ZNF320	1.248756	1.56E-09	6.83E-09
CLDN1	1.245879	5.44E-08	2.08E-07
FMC1	1.242411	0.000783	0.001866
GPR132	1.240263	2.32E-05	6.77E-05
FAAP24	1.238937	1.24E-07	4.56E-07
ZNF358	1.235902	3.96E-45	9.77E-44
PTPRN2	1.231816	1.63E-21	1.53E-20
H4C8	1.231591	0.000436	0.001079
BAHCC1	1.230231	1.76E-33	2.87E-32
BIRC3	1.219634	1.14E-32	1.80E-31
NUTM2E	1.214981	6.05E-11	2.97E-10
KANSL1L	1.210885	4.66E-06	1.47E-05
FAM210B	1.206398	7.40E-49	2.07E-47
FAM222A	1.200782	2.13E-10	9.99E-10
TMEM71	1.200383	8.12E-30	1.15E-28
AMOT	1.199938	8.70E-16	5.92E-15
TP53INP2	1.196887	7.15E-51	2.14E-49
TCP11L2	1.195856	0.000116	0.000311
LDB2	1.194689	5.45E-08	2.08E-07
SYNJ2BP-COX16	1.194668	1.63E-06	5.36E-06
SLC35A5	1.189951	1.86E-53	6.08E-52
DNAH10	1.18968	3.86E-05	0.00011
PITPNC1	1.187354	1.87E-37	3.59E-36
LRRC27	1.186956	3.28E-05	9.40E-05
TNFRSF8	1.185111	0.000477	0.001172
FAM156A	1.179727	3.12E-26	3.73E-25
ADRB2	1.177561	3.56E-62	1.54E-60
PGPEP1	1.177143	1.21E-06	4.02E-06
FGFBP3	1.174377	3.46E-05	9.89E-05
IFI27L1	1.174109	8.17E-07	2.76E-06
C8orf82	1.170652	3.78E-35	6.59E-34
GPX3	1.170326	3.94E-76	2.32E-74
ACAD10	1.169591	3.73E-16	2.61E-15
MYO7A	1.167554	0.000126	0.000336
POLR2J3	1.166517	1.05E-52	3.26E-51
OASL	1.164455	3.42E-38	6.80E-37
EMILIN2	1.164204	2.06E-05	6.06E-05
NR2C2AP	1.157224	2.59E-25	2.96E-24
QRICH2	1.156885	7.72E-05	0.000211
NAB1	1.15635	1.71E-36	3.18E-35
ACOT1	1.149767	0.000193	0.000503
PLEKHG5	1.147739	0.000234	0.000603
SEMA3E	1.146406	2.00E-34	3.39E-33
KIF9	1.145562	1.39E-05	4.17E-05
ZNF558	1.144732	1.93E-24	2.09E-23

RSPH3	1.144281	9.54E-13	5.28E-12
DNAH1	1.144166	8.54E-11	4.13E-10
CDPF1	1.143744	3.54E-07	1.25E-06
CCDC24	1.141786	3.42E-07	1.21E-06
TEF	1.138365	1.31E-09	5.79E-09
FRMD3	1.137443	1.30E-46	3.36E-45
TMEM14A	1.136914	1.26E-23	1.32E-22
PKIA	1.135796	1.09E-15	7.32E-15
DENND3	1.135348	1.23E-40	2.66E-39
PTPN6	1.134296	2.09E-12	1.13E-11
PTPN18	1.134171	3.90E-35	6.79E-34
PIGV	1.133926	1.26E-17	9.64E-17
QPCT	1.133357	5.61E-27	6.93E-26
SDSL	1.129483	1.03E-28	1.39E-27
CCL28	1.12604	0.000306	0.000774
VEGFC	1.125139	4.82E-27	5.98E-26
SH2B1	1.123031	6.81E-42	1.53E-40
RABL2B	1.122629	6.01E-14	3.59E-13
GLIPR1	1.121226	2.87E-33	4.64E-32
TCN2	1.117081	2.43E-11	1.22E-10
CARD6	1.114786	6.04E-16	4.17E-15
KYNU	1.113428	2.05E-48	5.67E-47
STEAP1	1.1121	4.13E-27	5.13E-26
ADAM8	1.111984	8.59E-38	1.68E-36
BMAL2	1.109184	5.12E-39	1.06E-37
APH1B	1.108839	0.000222	0.000575
MYOM3	1.105623	5.98E-32	9.31E-31
AKAP6	1.104589	2.20E-06	7.15E-06
PTCD1	1.098155	3.62E-07	1.27E-06
STK11IP	1.094649	1.04E-28	1.41E-27
GCLC	1.091342	2.47E-44	5.96E-43
KAZALD1	1.090831	8.76E-08	3.28E-07
JRK	1.090719	3.45E-67	1.68E-65
YJU2	1.087938	3.87E-35	6.75E-34
RASA4B	1.087671	3.15E-06	1.01E-05
MMP19	1.086983	5.70E-08	2.17E-07
MISP	1.086301	2.67E-31	4.03E-30
HDAC5	1.085923	1.49E-34	2.55E-33
GSTZ1	1.082813	1.33E-34	2.28E-33
ITGBL1	1.081358	8.19E-16	5.57E-15
DMAC1	1.079748	1.04E-21	9.78E-21
DXO	1.077961	2.65E-10	1.23E-09
SLC25A42	1.077777	6.99E-12	3.65E-11
GPAT2	1.077228	2.14E-55	7.52E-54
POLR2J3	1.076692	1.59E-13	9.25E-13
PARD3B	1.07598	8.20E-17	5.97E-16
TMEM144	1.075312	5.35E-07	1.85E-06
TEN1	1.074887	1.51E-14	9.37E-14
MAML3	1.074597	6.30E-17	4.61E-16
ACTA2	1.074195	0.000493	0.001206

SLC46A1	1.07264	1.31E-23	1.37E-22
CASD1	1.067036	8.97E-29	1.22E-27
FRAS1	1.066173	1.40E-27	1.79E-26
IL7	1.062914	0.000223	0.000576
FAM200C	1.062302	8.43E-11	4.09E-10
PEX26	1.061909	9.98E-57	3.66E-55
SOBP	1.058628	1.82E-07	6.58E-07
CA11	1.056606	0.000173	0.000454
GPR153	1.056458	3.07E-15	2.00E-14
MAST1	1.053298	1.73E-17	1.31E-16
SELENOO	1.049039	4.91E-26	5.80E-25
ALKBH4	1.049022	6.15E-21	5.61E-20
ZSCAN21	1.049012	1.78E-09	7.74E-09
PSEN1	1.048591	6.40E-48	1.75E-46
ARSA	1.04788	2.07E-09	9.00E-09
PGLS	1.04691	1.17E-44	2.84E-43
IRAK2	1.046873	2.94E-27	3.69E-26
C4orf3	1.043113	2.53E-35	4.45E-34
ZBTB1	1.040195	1.38E-07	5.07E-07
GATD3	1.03507	1.32E-37	2.56E-36
DFFB	1.033702	0.000179	0.00047
MVP	1.033068	3.97E-57	1.49E-55
PMEPA1	1.031692	1.34E-07	4.91E-07
ULK4	1.031478	3.78E-05	0.000107
GATAD1	1.030757	1.59E-50	4.72E-49
ZNF219	1.030215	2.98E-15	1.95E-14
ZNHIT1	1.028632	6.05E-78	3.68E-76
CHKB	1.022274	4.32E-07	1.51E-06
KHDC1	1.021578	2.58E-09	1.11E-08
HOXB9	1.021196	1.21E-13	7.09E-13
C21orf58	1.019862	9.39E-09	3.83E-08
TRIM41	1.018235	1.49E-61	6.37E-60
B4GALT6	1.017383	3.21E-38	6.39E-37
DAW1	1.016779	0.000201	0.000523
CLBA1	1.016411	2.23E-10	1.04E-09
WWOX	1.016177	0.000904	0.002134
ITGA7	1.01427	3.61E-05	0.000103
TCTN1	1.014107	1.01E-17	7.81E-17
FBXW4	1.013466	5.27E-25	5.92E-24
IFIT3	1.012369	4.80E-15	3.09E-14
SATB1	1.011762	4.89E-10	2.22E-09
FBXO43	1.011464	6.27E-15	4.00E-14
PRR16	1.011323	5.15E-10	2.34E-09
SLC39A3	1.011291	2.83E-19	2.39E-18
SPOCD1	1.010515	0.000484	0.001187
VGLL4	1.010428	1.31E-40	2.84E-39
MAN2C1	1.008802	1.64E-41	3.64E-40
VARS2	1.004901	1.97E-20	1.76E-19
CMC4	1.00094	7.33E-05	0.000201
PDGFA	-1.00022	3.59E-19	3.02E-18

TRIM6	-1.00072	0.000137	0.000364
BCL9	-1.00143	4.46E-35	7.74E-34
IL17RD	-1.00212	3.14E-21	2.91E-20
TBK1	-1.00311	3.77E-56	1.35E-54
DDIT4	-1.00431	3.03E-91	2.36E-89
UBE2V2	-1.00441	1.06E-39	2.24E-38
GPR160	-1.00445	4.35E-07	1.52E-06
ZNF420	-1.00499	1.31E-07	4.82E-07
FAM135A	-1.00662	2.18E-11	1.10E-10
LYSMD1	-1.00765	0.000512	0.001251
LTK	-1.00811	0.00092	0.002168
PLXNB3	-1.00948	1.98E-08	7.81E-08
GREB1	-1.00963	1.56E-06	5.15E-06
MSH5-SAPCD1	-1.00967	2.03E-05	5.98E-05
SEC62	-1.01059	3.48E-53	1.12E-51
TEAD2	-1.01077	1.77E-21	1.66E-20
SH3BP1	-1.01192	4.31E-20	3.77E-19
BTBD19	-1.01298	0.000795	0.001891
TAX1BP1	-1.01382	1.10E-53	3.64E-52
SNX27	-1.01418	3.05E-32	4.78E-31
LMX1B	-1.01441	4.98E-06	1.56E-05
ATP2B4	-1.01546	1.16E-65	5.50E-64
FAM217B	-1.01684	1.38E-09	6.07E-09
TDRKH	-1.01792	1.54E-28	2.05E-27
NBPF10	-1.01832	1.85E-29	2.59E-28
ZNF883	-1.01963	0.000177	0.000465
CENPL	-1.02032	1.63E-16	1.17E-15
SEMA3G	-1.02166	2.13E-05	6.26E-05
TMF1	-1.0223	1.67E-32	2.63E-31
FST	-1.02264	5.77E-07	1.99E-06
USPL1	-1.0228	3.32E-12	1.77E-11
FSBP	-1.02299	9.87E-05	0.000267
CLDN4	-1.02351	1.75E-33	2.85E-32
FOXN2	-1.02539	4.99E-23	5.07E-22
TBC1D15	-1.02556	9.81E-25	1.08E-23
PLEKHO1	-1.02578	2.86E-13	1.64E-12
MOSPD2	-1.0266	7.25E-16	4.96E-15
SCFD1	-1.02665	3.30E-51	9.96E-50
WWC1	-1.02772	1.90E-28	2.53E-27
HSPA1A	-1.02859	1.56E-06	5.14E-06
DUSP10	-1.03041	0.000104	0.000282
SLC37A2	-1.03133	0.000104	0.000282
USP25	-1.03165	1.46E-25	1.69E-24
RPAP3	-1.03338	7.88E-28	1.02E-26
CFH	-1.03369	3.34E-12	1.78E-11
CXXC5	-1.03435	9.05E-23	9.08E-22
ZHX1	-1.03444	2.45E-27	3.09E-26
ABCA5	-1.03599	4.23E-07	1.48E-06
GOLIM4	-1.03644	3.18E-37	6.07E-36
LBR	-1.03711	1.04E-87	7.68E-86

SERTAD4	-1.03969	2.39E-17	1.80E-16
B4GALNT1	-1.0422	1.48E-10	7.02E-10
PDE7A	-1.04576	2.87E-17	2.15E-16
ALMS1	-1.04638	2.17E-41	4.81E-40
ENAH	-1.04738	5.71E-86	4.16E-84
CEP70	-1.0482	2.14E-16	1.52E-15
BCKDHA	-1.05047	5.36E-13	3.02E-12
RASSF2	-1.05121	4.35E-18	3.46E-17
DNAJB11	-1.05633	3.20E-55	1.12E-53
ZEB2	-1.05654	2.78E-38	5.57E-37
NEURL1B	-1.05694	2.70E-17	2.02E-16
CCDC66	-1.05744	1.49E-11	7.61E-11
INO80B-WBP1	-1.05971	0.000255	0.000653
GK5	-1.0619	7.04E-18	5.51E-17
USP8	-1.06192	6.83E-33	1.09E-31
TOR1AIP1	-1.06247	8.22E-59	3.24E-57
MBNL2	-1.06261	3.40E-53	1.09E-51
TENT5B	-1.06432	6.59E-06	2.04E-05
ZNF845	-1.06474	6.88E-06	2.13E-05
CHD7	-1.06939	5.05E-39	1.04E-37
FAM72D	-1.06984	4.67E-14	2.82E-13
RSC1A1	-1.07048	1.52E-06	5.00E-06
ZNF296	-1.07076	6.08E-06	1.89E-05
ZNF225	-1.07719	2.98E-06	9.58E-06
FANCA	-1.07833	2.10E-26	2.53E-25
LEMD3	-1.0801	6.81E-37	1.29E-35
BTBD8	-1.08025	0.000132	0.000351
ZBED6	-1.08187	1.53E-64	6.98E-63
DNAH14	-1.08248	2.41E-06	7.82E-06
ADGRA2	-1.08532	2.14E-06	6.98E-06
TRIM24	-1.08586	8.60E-33	1.37E-31
IL7R	-1.0873	9.57E-98	8.33E-96
CKAP2	-1.08799	1.45E-77	8.77E-76
CALML4	-1.08825	1.20E-07	4.41E-07
NBPF14	-1.08888	9.83E-76	5.78E-74
MATN3	-1.09035	0.000133	0.000356
RND3	-1.09139	1.28E-38	2.61E-37
CMIP	-1.09277	5.00E-52	1.53E-50
MDM1	-1.09311	1.02E-14	6.38E-14
CHD9	-1.09339	9.40E-29	1.28E-27
TCF7L1	-1.09384	6.31E-10	2.84E-09
ARID5B	-1.09391	4.60E-10	2.10E-09
GATA2	-1.09477	2.31E-25	2.65E-24
ZMYM4	-1.09529	1.03E-36	1.93E-35
PRKAB2	-1.0954	1.51E-22	1.50E-21
ZNF613	-1.09642	0.000148	0.000394
SH3D21	-1.09876	4.34E-13	2.47E-12
SKP2	-1.09889	4.84E-61	2.02E-59
NUP107	-1.10048	2.31E-68	1.16E-66
ZFC3H1	-1.10091	2.87E-44	6.90E-43

MAGEA2B	-1.10562	2.58E-09	1.11E-08
TIAM2	-1.10613	6.82E-10	3.06E-09
NBPF11	-1.10619	1.43E-16	1.03E-15
ARHGAP40	-1.10646	2.04E-16	1.45E-15
NR1H3	-1.10744	3.16E-16	2.22E-15
CXorf38	-1.10746	4.41E-25	4.97E-24
GPRC5C	-1.10912	1.12E-07	4.15E-07
CPSF3	-1.11009	8.28E-48	2.25E-46
VPS54	-1.11016	5.63E-27	6.96E-26
GATA3	-1.11205	4.06E-08	1.56E-07
PIMREG	-1.11304	5.84E-43	1.35E-41
FAM241B	-1.1139	2.16E-05	6.33E-05
ARMC8	-1.11428	1.42E-25	1.65E-24
TTC27	-1.11798	3.92E-29	5.42E-28
EVI2B	-1.11927	4.73E-11	2.33E-10
SIPA1L2	-1.12473	8.49E-08	3.18E-07
LRP8	-1.12548	1.83E-57	6.89E-56
DLG1	-1.1257	1.14E-49	3.28E-48
TADA1	-1.12651	6.85E-16	4.70E-15
KMT5C	-1.1288	1.64E-06	5.40E-06
TAF1B	-1.13077	9.54E-10	4.25E-09
TARS3	-1.13566	6.18E-06	1.92E-05
DEF8	-1.13684	1.85E-22	1.82E-21
ZBTB46	-1.13742	1.34E-15	8.97E-15
GPLD1	-1.13998	0.000725	0.001739
PLOD2	-1.14144	2.49E-72	1.39E-70
ZNF251	-1.14152	3.69E-13	2.11E-12
CLCN2	-1.14475	6.06E-12	3.18E-11
RB1CC1	-1.14551	3.77E-22	3.63E-21
NEK7	-1.14681	3.99E-72	2.22E-70
SLC39A10	-1.14998	5.66E-83	3.79E-81
CDC42BPA	-1.15039	5.80E-84	4.05E-82
RNF207	-1.15393	2.61E-06	8.44E-06
VDR	-1.15515	3.01E-31	4.53E-30
TICAM2	-1.15522	2.43E-07	8.70E-07
SMAD6	-1.15539	6.77E-23	6.84E-22
TUBB3	-1.15613	4.91E-31	7.33E-30
RTEL1-	-1.15739	0.000524	0.001278
XAGE1B	-1.1595	1.13E-16	8.16E-16
NMD3	-1.16582	2.95E-54	9.95E-53
IGSF8	-1.16925	7.66E-28	9.90E-27
SFT2D2	-1.16935	4.48E-46	1.14E-44
MAPK8IP1	-1.17042	1.98E-10	9.27E-10
NACC2	-1.17131	2.42E-60	1.00E-58
MED12L	-1.17208	7.20E-08	2.72E-07
DBNDD1	-1.17313	1.46E-11	7.48E-11
TMEFF1	-1.17585	0.000539	0.001312
FUT1	-1.17585	0.00019	0.000496
FRS2	-1.17771	3.50E-46	8.94E-45
ZNF182	-1.18045	9.94E-06	3.02E-05

CABLES2	-1.18445	6.48E-33	1.04E-31
WNT10B	-1.18673	0.000899	0.002125
SRGAP2C	-1.18776	5.45E-65	2.53E-63
SORBS1	-1.19008	0.000742	0.001776
ZNF513	-1.19311	6.17E-11	3.03E-10
ARHGAP11A	-1.19424	1.86E-83	1.27E-81
PARD6G	-1.19462	1.67E-06	5.49E-06
ENOX1	-1.19486	2.99E-07	1.06E-06
SELENBP1	-1.19665	4.56E-16	3.18E-15
ST3GAL5	-1.19796	3.18E-21	2.95E-20
NBPF9	-1.20003	5.67E-83	3.79E-81
EPAS1	-1.20086	6.52E-54	2.17E-52
FAM156B	-1.20114	1.93E-49	5.47E-48
STRN3	-1.20146	1.21E-62	5.32E-61
CCDC28B	-1.20166	0.000349	0.000876
SOX13	-1.20318	1.26E-33	2.08E-32
DEPDC1	-1.20506	1.19E-82	7.85E-81
CREG1	-1.20744	1.04E-59	4.24E-58
TINAGL1	-1.20972	2.51E-54	8.51E-53
PCBP3	-1.20975	2.69E-06	8.67E-06
PLEKHG1	-1.20997	0.000344	0.000864
RNF122	-1.21538	0.000162	0.000427
SUSD3	-1.21666	8.89E-17	6.47E-16
CYP1B1	-1.21795	1.13E-46	2.92E-45
PACSIN3	-1.22002	2.04E-40	4.40E-39
B3GALNT1	-1.22113	6.83E-34	1.13E-32
SEMA6C	-1.22239	0.000227	0.000585
ZNF850	-1.22704	1.00E-13	5.92E-13
PLCH1	-1.22741	1.46E-17	1.11E-16
FREM2	-1.23138	1.72E-17	1.31E-16
RASSF3	-1.2317	1.05E-64	4.84E-63
MTARC1	-1.23172	2.20E-17	1.66E-16
PI15	-1.23212	0.000118	0.000318
MFAP3L	-1.23365	1.08E-05	3.26E-05
XPR1	-1.23418	5.42E-67	2.63E-65
SPIRE2	-1.23566	8.45E-14	5.00E-13
DNAJB4	-1.2369	1.70E-39	3.57E-38
RLF	-1.23718	2.26E-34	3.82E-33
MARCKSL1	-1.23718	4.95E-60	2.03E-58
TBC1D30	-1.24089	5.62E-21	5.14E-20
IQCC	-1.24149	3.46E-05	9.88E-05
MPHOSPH10	-1.24322	6.10E-32	9.48E-31
CHD1	-1.24334	6.13E-50	1.79E-48
IL12A	-1.24417	0.000389	0.000969
SARDH	-1.24528	5.00E-13	2.83E-12
FLVCR1	-1.24619	2.34E-26	2.82E-25
C16orf87	-1.24671	2.20E-12	1.18E-11
PTGFRN	-1.25086	4.34E-57	1.62E-55
CHST2	-1.25226	6.41E-18	5.03E-17
SCCPDH	-1.25409	5.02E-43	1.17E-41

RASA2	-1.25467	1.27E-19	1.09E-18
ZNF232	-1.25553	4.88E-05	0.000137
BBC3	-1.26368	0.000221	0.000573
OPLAH	-1.2684	3.97E-13	2.26E-12
FIBCD1	-1.26939	1.38E-06	4.56E-06
MAGEE1	-1.27053	8.94E-09	3.65E-08
ADAMTS7	-1.27127	5.41E-10	2.45E-09
KCNQ4	-1.2717	1.10E-10	5.30E-10
SLC35F3	-1.27187	1.04E-10	5.03E-10
DENND1B	-1.27204	6.75E-05	0.000186
GNAO1	-1.27498	1.08E-14	6.78E-14
KCNN4	-1.27669	2.01E-30	2.94E-29
PPFIA4	-1.28112	9.23E-07	3.11E-06
LHX4	-1.28146	2.69E-06	8.69E-06
TMOD2	-1.28255	7.76E-17	5.66E-16
PITPNM3	-1.28492	1.52E-05	4.53E-05
SDR16C5	-1.28862	6.71E-07	2.29E-06
POMT1	-1.29186	1.10E-31	1.69E-30
MARK4	-1.29296	1.29E-35	2.29E-34
ANOS1	-1.29793	0.000388	0.000966
GXYLT1	-1.29948	8.68E-53	2.74E-51
F13A1	-1.30472	1.43E-15	9.55E-15
FKBP11	-1.30526	0.000274	0.000699
MAP6D1	-1.31251	3.10E-11	1.55E-10
CPQ	-1.31581	0.000181	0.000474
ZNF239	-1.3204	1.45E-09	6.36E-09
TRAF5	-1.32242	5.18E-21	4.75E-20
FAM174B	-1.32478	3.37E-10	1.56E-09
ZNF649	-1.32644	0.000431	0.001067
BICC1	-1.33054	1.12E-19	9.61E-19
PIK3CA	-1.33262	7.58E-32	1.17E-30
G2E3	-1.33669	7.93E-39	1.62E-37
ANGPTL2	-1.33868	5.87E-11	2.88E-10
TMEM150C	-1.34264	9.28E-07	3.12E-06
IDH2	-1.3482	4.42E-47	1.17E-45
MAMDC2	-1.34831	0.000114	0.000306
ZNF468	-1.35089	1.41E-13	8.25E-13
CREB5	-1.35255	6.81E-16	4.68E-15
FBLIM1	-1.35346	1.22E-36	2.28E-35
TOX	-1.35359	1.71E-14	1.06E-13
ARHGAP9	-1.35391	0.000223	0.000576
SFXN2	-1.35481	2.51E-05	7.29E-05
PCCA	-1.35693	4.62E-08	1.77E-07
ZNF254	-1.36009	7.11E-06	2.20E-05
H4C15	-1.36147	2.27E-05	6.64E-05
FN3K	-1.36194	6.72E-05	0.000185
SLC25A22	-1.36468	2.22E-61	9.34E-60
ROR1	-1.36572	5.56E-35	9.62E-34
LPL	-1.36576	0.000543	0.001322
TSPAN33	-1.37977	7.01E-11	3.42E-10

PPP1R3D	-1.38196	4.68E-05	0.000132
OTULINL	-1.38541	1.60E-17	1.22E-16
ARID4B	-1.39248	2.69E-27	3.39E-26
IGFBP2	-1.40577	1.20E-08	4.82E-08
CNTN1	-1.40896	0.000298	0.000754
VWA1	-1.40981	3.77E-19	3.16E-18
LIFR	-1.41195	1.01E-50	3.02E-49
ZNF124	-1.41755	2.43E-08	9.53E-08
DNAJC6	-1.41773	1.75E-49	4.99E-48
BEX2	-1.42364	1.13E-05	3.39E-05
IFITM2	-1.42389	9.79E-84	6.81E-82
THUMPD2	-1.43005	7.34E-19	6.06E-18
TMEM108	-1.43431	3.07E-23	3.16E-22
ASPM	-1.43748	9.53E-62	4.10E-60
SMIM10	-1.43763	1.36E-06	4.51E-06
NOTCH2NLC	-1.43859	4.35E-15	2.81E-14
FAM72C	-1.43912	1.14E-28	1.54E-27
LRP2	-1.44057	0.000917	0.002163
GDAP1	-1.44057	7.13E-44	1.70E-42
NNMT	-1.44125	0.000507	0.001238
ASS1	-1.44869	1.00E-85	7.27E-84
PALM	-1.44983	8.43E-17	6.14E-16
SNAI1	-1.45136	4.21E-10	1.92E-09
PUSL1	-1.45339	5.36E-20	4.66E-19
KLHL4	-1.46093	5.32E-20	4.63E-19
VIPR1	-1.46672	3.86E-13	2.20E-12
NANOS1	-1.47747	4.91E-12	2.59E-11
VASH2	-1.47779	8.44E-06	2.58E-05
ALDH5A1	-1.48078	1.50E-19	1.28E-18
SH3PXD2B	-1.4841	5.99E-56	2.13E-54
TLR4	-1.48454	3.12E-29	4.33E-28
LRP4	-1.49612	1.00E-28	1.36E-27
ATR	-1.49849	1.49E-61	6.37E-60
UCP2	-1.49905	9.09E-92	7.12E-90
PARD6B	-1.50028	1.80E-10	8.47E-10
GPR75	-1.50173	1.31E-07	4.81E-07
ARHGEF6	-1.50535	3.10E-08	1.21E-07
NOTCH2NLB	-1.50645	3.57E-21	3.30E-20
BLZF1	-1.50742	2.42E-19	2.05E-18
SYNM	-1.51008	5.43E-57	2.01E-55
SUCO	-1.51901	1.53E-64	6.98E-63
BSCL2	-1.5271	2.70E-13	1.55E-12
SH2D2A	-1.52864	9.01E-05	0.000245
KIF14	-1.52873	2.67E-67	1.30E-65
BMP8B	-1.52897	8.72E-19	7.18E-18
KCNIP3	-1.53255	5.20E-06	1.63E-05
COLGALT2	-1.53348	4.56E-11	2.25E-10
ZNF547	-1.53379	6.18E-05	0.000171
DYNC1I1	-1.5386	8.16E-08	3.07E-07
S100A4	-1.53968	3.39E-36	6.18E-35

PPP1R12B	-1.54006	6.79E-15	4.31E-14
PDP2	-1.5605	1.01E-13	5.96E-13
RAB6B	-1.56397	1.39E-24	1.52E-23
CPE	-1.56732	5.24E-19	4.35E-18
TMC8	-1.58701	9.91E-18	7.67E-17
ESYT3	-1.59007	9.00E-05	0.000245
RAB3A	-1.59156	1.76E-05	5.21E-05
MICALL2	-1.5927	2.96E-14	1.81E-13
SDC2	-1.59459	7.44E-55	2.57E-53
IL16	-1.59538	1.55E-06	5.10E-06
SH3TC1	-1.59748	5.63E-16	3.90E-15
HSBP1L1	-1.60024	3.08E-07	1.09E-06
HR	-1.60354	2.22E-15	1.46E-14
NOSTRIN	-1.60459	8.47E-05	0.000231
MRAS	-1.61682	7.89E-13	4.41E-12
CEP350	-1.61755	7.26E-60	2.97E-58
NPTXR	-1.62027	1.25E-08	5.05E-08
TFAP2C	-1.62353	6.43E-67	3.11E-65
AARD	-1.6291	1.35E-06	4.50E-06
HPCAL4	-1.63472	2.14E-08	8.43E-08
HOOK1	-1.63786	8.34E-17	6.07E-16
CLIC3	-1.63947	3.49E-05	9.96E-05
PCDH20	-1.64142	6.23E-21	5.68E-20
ZNF92	-1.64327	2.27E-11	1.15E-10
S100A3	-1.64468	3.45E-12	1.84E-11
PRICKLE1	-1.64713	1.25E-25	1.45E-24
CNTF	-1.65208	0.000477	0.001172
EFCAB2	-1.6555	0.000761	0.001817
AQP1	-1.66367	7.56E-06	2.33E-05
HES1	-1.67753	4.25E-13	2.42E-12
CDK5R1	-1.67973	1.28E-12	7.04E-12
PARM1	-1.6909	0.000239	0.000616
HECW2	-1.69599	5.56E-10	2.51E-09
SEC14L6	-1.69637	5.19E-06	1.63E-05
ADORA1	-1.70008	3.84E-11	1.91E-10
SETBP1	-1.70402	0.000173	0.000454
SDK1	-1.70501	8.75E-18	6.80E-17
PPFIBP2	-1.70644	3.36E-09	1.43E-08
ENPP2	-1.71912	1.35E-05	4.04E-05
ARHGAP11B	-1.72374	6.95E-06	2.15E-05
SIX2	-1.74358	3.70E-07	1.30E-06
CLCN5	-1.75137	4.28E-20	3.74E-19
RGPD5	-1.75466	2.61E-17	1.96E-16
HIP1	-1.78224	2.26E-98	1.98E-96
PXYLP1	-1.78524	1.01E-15	6.84E-15
ERO1B	-1.78557	6.66E-17	4.87E-16
TIMM23B-AGAP6	-1.79222	2.25E-08	8.88E-08
JPH2	-1.8027	4.33E-05	0.000122
ESRP2	-1.8033	4.73E-18	3.75E-17
COL7A1	-1.81807	6.78E-73	3.84E-71

EML5	-1.82049	4.97E-09	2.08E-08
DYSF	-1.83132	3.99E-19	3.34E-18
AGMAT	-1.83153	1.83E-14	1.13E-13
CRISPLD2	-1.8365	0.000103	0.000278
RALGPS2	-1.83877	2.49E-94	2.07E-92
FOXF2	-1.84072	6.85E-27	8.43E-26
PTK7	-1.84077	1.62E-69	8.30E-68
EFNB3	-1.84543	2.71E-09	1.16E-08
HTRA3	-1.85099	9.70E-07	3.26E-06
IER5L	-1.85188	1.38E-34	2.36E-33
MAFA	-1.85277	2.06E-06	6.72E-06
TP73	-1.862	1.74E-22	1.71E-21
SEMA6A	-1.86546	6.11E-13	3.43E-12
CNIH3	-1.87248	1.35E-09	5.92E-09
TSPAN2	-1.87553	2.65E-15	1.74E-14
TCEA3	-1.87762	1.55E-20	1.39E-19
CREB3L1	-1.87885	9.82E-80	6.27E-78
MATN2	-1.88638	2.01E-16	1.43E-15
DAAM2	-1.90617	4.74E-25	5.34E-24
CORO1A	-1.90873	1.07E-07	3.95E-07
NEDD9	-1.90979	1.54E-25	1.78E-24
LOXL4	-1.91228	4.82E-05	0.000135
OSR1	-1.91321	6.00E-09	2.50E-08
CSPG4	-1.9144	1.35E-80	8.69E-79
SYT13	-1.91754	5.95E-12	3.12E-11
MOB3B	-1.9367	3.09E-05	8.88E-05
SIGIRR	-1.94108	0.00075	0.001794
EPHB3	-1.94596	3.67E-05	0.000104
OR2M4	-1.94698	3.67E-05	0.000104
KLRC2	-1.94727	1.11E-13	6.55E-13
ILDR2	-1.9486	1.26E-13	7.38E-13
MUC15	-1.95441	6.42E-07	2.20E-06
PNPLA3	-1.956	9.54E-12	4.95E-11
RHOU	-1.97342	5.89E-14	3.53E-13
SLC43A2	-1.97431	7.60E-31	1.12E-29
MGAT5B	-1.98128	2.09E-38	4.22E-37
SELENOM	-1.99406	1.65E-17	1.26E-16
NPIPB12	-2.00563	4.70E-11	2.32E-10
WASF3	-2.01804	1.98E-40	4.28E-39
GSDMB	-2.02917	0.000436	0.001079
SLC19A3	-2.03423	6.93E-19	5.72E-18
LIMD2	-2.04149	5.93E-05	0.000165
APOC1	-2.05627	4.28E-13	2.43E-12
COL9A3	-2.06616	1.42E-11	7.28E-11
HELB	-2.06922	3.12E-09	1.33E-08
FOXD2	-2.07549	0.000886	0.002095
FGFR3	-2.07781	2.48E-24	2.68E-23
PRR5	-2.08239	2.44E-05	7.10E-05
VPS35L	-2.09147	5.08E-17	3.74E-16
RHOV	-2.10891	6.69E-05	0.000185

FAM83H	-2.11186	6.02E-16	4.16E-15
PCED1B	-2.12342	1.08E-10	5.17E-10
VASN	-2.1265	2.02E-09	8.79E-09
ATP6V1C2	-2.13107	5.94E-07	2.05E-06
C4orf19	-2.13946	0.00012	0.000321
TENT5C	-2.14037	9.80E-52	2.99E-50
KCNS3	-2.14958	1.50E-21	1.41E-20
GABRE	-2.15877	3.91E-35	6.81E-34
KCNN3	-2.16398	3.90E-06	1.24E-05
LYL1	-2.17672	1.67E-07	6.06E-07
EFEMP1	-2.17936	1.30E-61	5.57E-60
ELMO1	-2.19184	1.31E-19	1.12E-18
MFNG	-2.19653	0.000761	0.001817
ADAMTS15	-2.20613	1.58E-39	3.32E-38
EAF2	-2.2158	0.000919	0.002168
ATP10A	-2.23672	4.70E-13	2.67E-12
SLCO4C1	-2.23958	1.40E-22	1.39E-21
ADGRG2	-2.24793	6.06E-12	3.18E-11
ADM	-2.25811	2.25E-62	9.79E-61
CALHM3	-2.2821	1.50E-08	5.99E-08
PTGES	-2.28247	4.98E-72	2.76E-70
PTPRH	-2.29332	6.59E-08	2.49E-07
SCG2	-2.30053	1.57E-11	8.00E-11
WIPF3	-2.32923	2.96E-10	1.37E-09
NOS3	-2.33792	1.40E-36	2.61E-35
CD82	-2.35011	1.86E-71	1.01E-69
SHISAL1	-2.35712	3.83E-20	3.37E-19
BTG2	-2.3644	1.87E-13	1.09E-12
FHDC1	-2.38042	1.08E-07	3.98E-07
ECM1	-2.38642	5.16E-89	3.98E-87
ZNF365	-2.3874	2.54E-05	7.39E-05
GAL3ST1	-2.40021	1.54E-05	4.60E-05
GRHL1	-2.40188	7.53E-07	2.56E-06
ZNF283	-2.40312	3.20E-06	1.02E-05
C10orf90	-2.40583	1.40E-19	1.20E-18
GYG2	-2.41489	1.23E-17	9.42E-17
STON1	-2.4164	3.15E-38	6.28E-37
AR	-2.4164	3.54E-48	9.71E-47
ADAMTSL1	-2.46964	3.48E-06	1.11E-05
KLRK1	-2.47685	3.91E-05	0.000111
RASGRF1	-2.48298	1.88E-22	1.85E-21
P2RY6	-2.50696	1.54E-05	4.59E-05
HAS2	-2.53417	1.58E-07	5.75E-07
BDKRB2	-2.54381	8.91E-09	3.64E-08
TDRP	-2.5575	3.05E-27	3.83E-26
CDK15	-2.56687	5.33E-38	1.06E-36
UCN2	-2.57176	3.23E-26	3.86E-25
CDH3	-2.59881	7.83E-27	9.61E-26
PDGFRB	-2.60098	3.78E-45	9.35E-44
NRARP	-2.62006	6.11E-13	3.43E-12

SDR42E1	-2.64715	5.77E-09	2.40E-08
CFAP45	-2.65437	1.97E-12	1.07E-11
RGS7	-2.65622	0.000917	0.002163
DLX2	-2.66034	0.000105	0.000283
HNF4G	-2.7028	0.000313	0.000791
GGT5	-2.71792	3.38E-66	1.62E-64
CPT1C	-2.72346	1.65E-21	1.55E-20
PSTPIP2	-2.73697	1.53E-23	1.59E-22
CEMIP	-2.75653	6.27E-88	4.67E-86
ABCA8	-2.79355	1.65E-09	7.19E-09
DUSP9	-2.79912	6.09E-26	7.17E-25
BST2	-2.79928	4.34E-06	1.37E-05
AGR2	-2.81416	3.91E-05	0.000111
NPY1R	-2.93631	4.22E-19	3.53E-18
SLC27A2	-2.94374	4.16E-08	1.60E-07
FAM133A	-2.95496	1.96E-05	5.77E-05
EPHA4	-2.96447	1.24E-37	2.40E-36
LYNX1	-2.974	0.000917	0.002163
L1CAM	-2.97487	1.18E-92	9.49E-91
CMYA5	-2.98185	0.000861	0.002038
ABI3	-2.9997	7.88E-26	9.24E-25
LZTS1	-3.01527	6.68E-15	4.25E-14
DMBX1	-3.05128	1.11E-05	3.35E-05
ATP8A2	-3.08179	0.000279	0.000711
SLC47A2	-3.09163	0.000279	0.000711
CAPS2	-3.11374	1.43E-07	5.24E-07
NELL2	-3.11623	1.21E-31	1.85E-30
CFP	-3.12353	1.49E-21	1.39E-20
SEMA6D	-3.1307	4.72E-06	1.48E-05
KAZN	-3.18531	1.70E-42	3.87E-41
DNAH6	-3.18587	0.000279	0.000711
STRA6	-3.24462	2.54E-08	9.96E-08
CA9	-3.24536	2.64E-20	2.34E-19
PHLDA3	-3.26679	7.41E-10	3.32E-09
SERPINF1	-3.29001	4.88E-20	4.26E-19
RAB37	-3.29598	5.92E-08	2.25E-07
PAK6	-3.31879	2.65E-13	1.53E-12
HMGN5	-3.36788	1.54E-05	4.59E-05
PLD1	-3.39734	1.15E-09	5.08E-09
DENND2A	-3.39763	3.52E-45	8.70E-44
ARHGAP30	-3.44683	7.63E-29	1.04E-27
PLCH2	-3.48843	4.84E-39	1.00E-37
ADAP2	-3.54703	4.80E-14	2.89E-13
NOTCH3	-3.57112	1.44E-08	5.77E-08
S100P	-3.57394	1.07E-13	6.30E-13
PADI1	-3.60306	2.60E-06	8.40E-06
CTTNBP2	-3.62449	0.000223	0.000576
TRPV2	-3.67101	6.69E-54	2.22E-52
KRT86	-3.79846	2.24E-10	1.05E-09
BEND4	-3.81569	4.78E-17	3.52E-16

ANKRD2	-3.82262	0.000405	0.001007
PCDHGA12	-3.86507	1.49E-13	8.70E-13
ADAMTSL4	-3.88839	1.73E-16	1.24E-15
LARGE2	-3.93256	0.000223	0.000576
TCF4	-3.94976	6.05E-09	2.52E-08
CLDN7	-3.96073	7.30E-18	5.70E-17
INAVA	-4.04198	1.08E-87	7.98E-86
LARGE1	-4.11548	5.11E-16	3.55E-15
SHH	-4.22176	3.28E-12	1.75E-11
IQGAP2	-4.23869	7.02E-15	4.45E-14
RCAN2	-4.33271	5.72E-06	1.79E-05
ZNF569	-4.3568	6.02E-12	3.16E-11
KISS1	-4.36343	1.68E-16	1.20E-15
PLCB2	-4.36379	1.21E-31	1.85E-30
ALDH1A3	-4.4028	5.40E-33	8.68E-32
AFAP1L2	-4.44857	1.97E-61	8.34E-60
SLCO2A1	-4.45533	6.66E-05	0.000184
FBXO6	-4.47644	2.23E-10	1.04E-09
PADI3	-4.47897	1.52E-34	2.60E-33
FMN2	-4.4818	1.65E-06	5.42E-06
RIPOR3	-4.58696	2.06E-39	4.32E-38
PSCA	-4.59067	5.45E-32	8.50E-31
CDH11	-4.69661	1.42E-08	5.67E-08
ANXA8	-4.75654	3.68E-22	3.54E-21
LPAR1	-4.79579	1.02E-82	6.73E-81
MPZL2	-4.80946	7.95E-14	4.72E-13
IGFN1	-4.84435	4.59E-37	8.73E-36
RYR2	-4.88101	5.36E-31	7.98E-30
NCALD	-5.069	1.21E-14	7.52E-14
PDZK1	-5.07241	3.21E-11	1.60E-10
TRIM59	-5.28881	1.46E-68	7.41E-67
USP2	-5.35133	0.000732	0.001755
CD226	-5.45943	0.000122	0.000327
PBX1	-5.47735	1.53E-17	1.17E-16
SAA1	-5.5299	2.80E-27	3.52E-26
OLFML3	-5.76453	4.58E-70	2.38E-68
THSD7B	-5.80735	7.45E-16	5.09E-15
TENM3	-5.96193	3.43E-15	2.23E-14
ECSCR	-6.13227	3.21E-21	2.98E-20
ST6GAL1	-6.23601	1.21E-22	1.21E-21
BMP5	-6.30264	4.55E-24	4.87E-23
ALPK2	-6.39613	1.67E-27	2.12E-26
TRPM6	-6.79442	1.54E-05	4.58E-05
FBN2	-6.82508	5.85E-77	3.51E-75
PAPPA	-6.83289	2.41E-07	8.63E-07
EHD3	-6.90689	0.000979	0.002299
SLITRK4	-6.90689	0.000123	0.000328
NLRP3	-7.11894	0.000979	0.002299
BIVM-ERCC5	-7.15987	0.000123	0.000328
POTEI	-7.19967	7.69E-06	2.37E-05

CDH5	-7.40088	0.000245	0.00063
GRIK4	-7.57743	6.14E-05	0.00017
SEPTIN3	-7.57743	1.54E-05	4.58E-05
CHST9	-7.63662	0.000979	0.002299
NAP1L2	-7.82655	0.000979	0.002299
UNC5D	-7.88874	2.20E-07	7.88E-07
RIMBP3B	-7.99435	2.20E-07	7.88E-07
ABLIM2	-8.12412	0.000123	0.000328
ADAMTS12	-8.21293	1.65E-84	1.18E-82
LDLRAD4	-8.29002	6.14E-05	0.00017
RASGEF1A	-8.3966	0.00049	0.001199
PADI4	-8.40939	0.000123	0.000328
PRKCQ	-8.53138	4.82E-07	1.68E-06
PTGS1	-8.59246	1.57E-08	6.25E-08
ZNF709	-8.65821	2.93E-10	1.36E-09
COL4A6	-8.67243	4.90E-14	2.95E-13
SPN	-8.79766	4.82E-07	1.68E-06
AVPR2	-9.09276	3.07E-05	8.83E-05
KCNJ12	-9.09276	2.03E-11	1.03E-10
C2CD6	-9.43879	4.18E-09	1.76E-08
COL4A5	-9.44915	1.56E-22	1.54E-21
SMIM31	-9.52748	4.82E-07	1.68E-06
KCTD4	-9.54882	3.04E-08	1.18E-07
PSTPIP1	-9.73978	3.04E-08	1.18E-07
ANXA8L1	-9.81698	4.18E-09	1.76E-08
FMOD	-10.0112	4.42E-16	3.08E-15
PPP1R14C	-10.3718	1.70E-15	1.13E-14
MAGEB1	-10.3783	5.34E-12	2.81E-11
ZNF28	-10.584	3.06E-22	2.95E-21
ASRGL1	-10.6439	2.03E-11	1.03E-10
SHISA2	-10.6484	1.14E-31	1.74E-30
IFNAR2-IL10RB	-10.8719	3.25E-30	4.69E-29
C15orf48	-10.9506	3.03E-08	1.18E-07
CLEC11A	-11.2192	2.98E-17	2.23E-16
ZG16B	-12.3112	3.06E-22	2.95E-21
MAGEA1	-12.7232	6.75E-58	2.57E-56

Table S2. Primers of the top 5 differential expression genes

Gene	NCBI ID	Orientation	Primer (5'-3')	Temperature
ACTB	NM_001101.5	Forward	CATGTACGTTGCTATCCAGGC	58.0°C
(Human)		Reverse	CTCCTTAATGTCACGCACGAT	58.0°C
BCL2A1	NM_001114735.2	Forward	AGTGCTACAAAATGTTGCGTTC	60.0°C
(Human)		Reverse	GGCAATTTGCTGTCGTAGAAGTT	60.6°C
WFDC3	NM_080614.2	Forward	GATCTGCCGAGACATTCCTAAG	60.0°C
(Human)		Reverse	GCAGCATTTCTTTACACCTGGA	60.8°C
PI3	NM_002638.4	Forward	CACGGGAGTTCCTGTTAAAGG	60.3°C
(Human)		Reverse	TCTTTCAAGCAGCGGTTAGGG	62.4°C
CLDN5	NM_001130861.1	Forward	CTCTGCTGGTTCGCCAACAT	62.8°C
(Human)		Reverse	CAGCTCGTACTTCTGCGACA	61.9°C
MMP1	NM_001145938.2	Forward	AAAATTACACGCCAGATTTGCC	60.0°C
(Human)		Reverse	GGTGTGACATTACTCCAGAGTTG	60.0°C
